# Neddylation blockade induces HIF-1α driven cancer cell migration via upregulation of ZEB1

**DOI:** 10.1038/s41598-020-75286-0

**Published:** 2020-10-23

**Authors:** Jun Bum Park, Jieun Seo, Jong-Wan Park, Yang-Sook Chun

**Affiliations:** 1 Department of Biomedical Science, Seoul National University College of Medicine, Seoul, 110-799 Korea; 2 Ischemic/Hypoxic Disease Institute, Seoul National University College of Medicine, Seoul, 110-799 Korea; 3 Department of Physiology, Seoul National University College of Medicine, 103 Daehak-ro, Jongno-gu, Seoul, 110-799 Korea

**Keywords:** Lung cancer, Metastasis, Drug development, Cell migration, Post-translational modifications, Epigenetics, Post-translational modifications, RNAi, Cancer, Molecular medicine

## Abstract

Neddylation is a process by which NEDD8 is covalently conjugated to target proteins by sequential enzymatic reaction. Its role in cancer cell migration has only been recently acknowledged. Previously in cancer cell migration, the epithelial to mesenchymal transition (EMT) process has been well-known to play an important role in both invasion and metastasis by promoting mesenchymal phenotype in epithelial cells. However, the role of neddylation in the EMT process and its mechanistic details are yet to be elucidated. We recently reported that neddylation plays a crucial role in cancer cell migration through the PI3K-Akt pathway. Here, we report that inhibiting neddylation activates the hypoxia-inducible factor 1α (HIF-1α) through the PI3K-Akt pathway, which eventually regulates the EMT-activator ZEB1 (zinc finger E-box binding homeobox 1) in various cancer cell lines. As induction of HIF-1α is known to deteriorate the state of cancer and EMT process is one of the hallmarks of metastasis in cancer, our findings uncover the role of neddylation between HIF-1α and ZEB1.

## Introduction

Neddylation, a post translational modification comparable with ubiquitination, is involved in variety of human cancers. Neddylation goes through a process in which ubiquitin-like molecule NEDD8 regulates cullin-RING ubiquitin ligases (CRL) constituting E3 enzymes. Specifically, NEDD8-activating enzyme-E1 (NAE1), a heterodimer composed of ubiquitin-like modifier activating enzyme 3 (UBA3) and amyloid precursor protein-binding protein (APPBP1), is delivered to various NEDD8-E3 ligases by the E2 enzyme UBC12^1^. As a component of CRLs, cullin has been reported to be neddylated with specific roles in ubiquitin transfer activity^2^. Moreover, many non-cullin neddylation substrates have been implicated in significant processes regarding cancer. For instance, neddylation has been reported to regulate transcriptional activity of the tumor suppressor protein p53 through Mdm2^3^. Also, NFκB-dependent transcription in breast cancer is known to be involved in neddylation mediated by breast cancer-associated protein 3 (BCA3)^4^. As such, the elucidation of neddylation pathway in cancer shows strong potential as a promising treatment.

Recently, neddylation has been reported to be involved in metastasis^5^. Among many possible causes of metastasis, the epithelial–mesenchymal transition (EMT) is the primary mechanism by which epithelial cells obtain mesenchymal traits, triggering cell migration^6^. In many cases, EMT promoting transcription factors such as Slug, Snail, and zinc finger E-box binding homeobox1 (ZEB1) have been reported as manipulative players from cancer progression to metastasis^7^. In particular, ZEB1 is known for its high inverse correlation with E-cadherin in multiple carcinomas^8^. Also, high expression of ZEB1 is reported to be associated to poor patient survival and active metastasis^9^. In fact, ZEB1 is known to be activated by hypoxia inducible factor 1-alpha (HIF-1α), a master transcriptional regulator of many responses in tumor invasion^10^. In addition, ZEB1 is involved in one of the most common oncogenic pathway, PI3K/Akt/mTOR pathway^11^. Such involvement of ZEB1 led to the investigation of its role in cancer, which clarified transcriptional regulation of target genes and other types of epigenetic regulation involving post translational modification^12^. Nevertheless, unknown regulations regarding ZEB1 in cancer activity have yet to be elucidated.

Herein, based on previous reports regarding PI3K/Akt/mTOR pathway regulating HIF-1α and HIF-1α promoting Zeb1 expression^10,13^, we investigated the possible role of neddylation between HIF-1α and ZEB1 regarding cancer cell migration. Using MLN4924, we show that neddylation blockade induces ZEB1 expression. Furthermore, we find that ZEB1 induction is due to the activation of PI3K/Akt/mTOR pathway, in which HIF-1α acting as the mediator. Thus, this study states the possible involvement of neddylation as an indispensable modification in cancer metastasis.

## Results

### Impairment in neddylation induces migration in cancer cells

**Figure 1:**
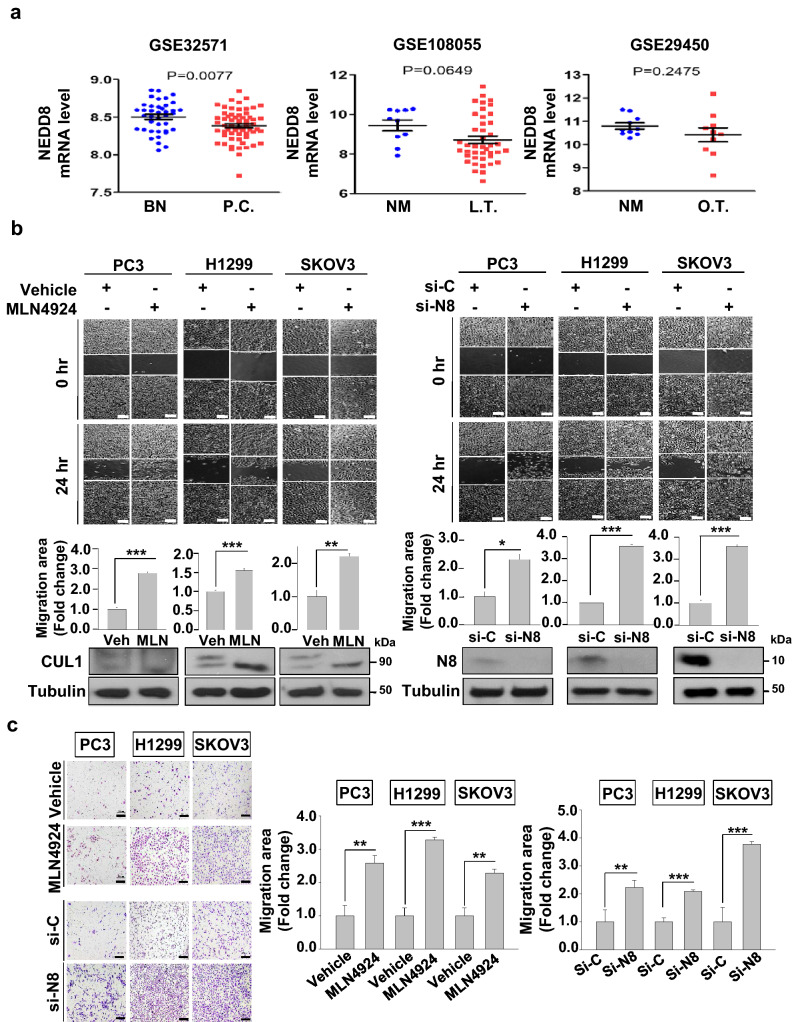
Impairment in neddylation induces migration in cancer cells. (**a**) The distribution of NEDD8 expression within normal (blue circles) and cancerous tissues (red squares) of lung (NEDD8_at probe), prostate (ILMN_1785711_at probe) and ovarian (201840_at probe). (**b**) PC3, H1299, and SKOV3 cells were subjected to wound healing assay with or without MLN4924 (0.125 μM) or transfected with si-Control (si-C) or si-NEDD8 (si-N8) in serum-free media as described in the method section. MLN4924 treated cells were harvested after 24 h of treatment while siRNA transfected cells were harvested 48 h after transfection. Lysates were then subjected to Western Blotting with the indicated antibodies. Scale bar: 200 μm. Empty areas were quantified using ImageJ software and data are presented as the means ± SD (n = 3). (**c**) PC3, H1299, and SKOV3 cells were subjected to Transwell migration assay with or without MLN4924 (0.125 μM) or transfected with si-Control (si-C) or si-NEDD8 (si-N8). The numbers of cells in four randomly chosen fields were counted. *p < 0.05; **p < 0.01; ***p < 0.001; *ns* not significant.

To identify the role of NEDD8 in cancer progression, we examined NEDD8 mRNA expressions in three different types of cancers. We specifically compared normal and cancer tissues for prostate, lung, and ovarian cancers. NEDD8 mRNA levels in prostate cancer significantly decreased while those of lung tumor and ovarian tumor did not (Fig. 1a). However, there was a declining tendency in NEDD8 mRNA levels of lung tumor tissues (*p* = 0.0649). Owing to the small sample size of the ovarian tumor tissues, we examined the effect of neddylation in cell migration for all three cancer cell lines. As we previously discovered the role of neddylation in c-Src for cancer cell migration^14^, we used the condition equivalent to our previous data, consisting of a 24 h treatment with 0.125 μM MLN4924 (Supplementary Figure S1, Supplementary Figure S2). Using PC3 (prostate), H1299 (lung), and SKOV3 (ovarian) cancer cell lines, we assessed cell migration via both wound healing assay and transwell assay by using MLN4924 and NEDD8-targeting siRNA (si-NEDD8). As a result, neddylation blockade significantly induced cell migration in PC3, H1299 and SKOV3 cancer cell lines (Fig. 1b,c). Induction in migration was further confirmed using a different siRNA targeting NEDD8 (Supplementary Figure S3). Therefore, these results imply the significant role of neddylation in regulating cell migration in variety of cancer cell lines.

### Blockage of neddylation regulates ZEB1

**Figure 2:**
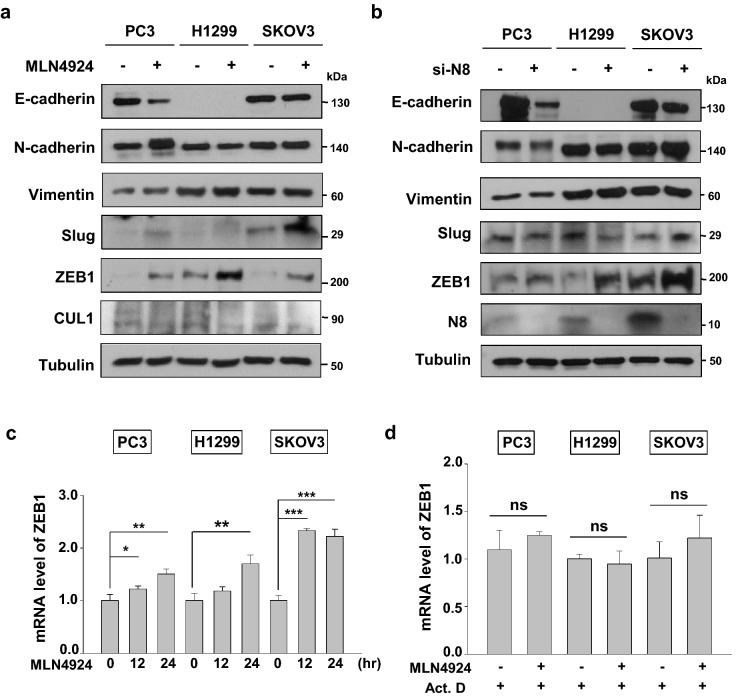
Blockage of neddylation regulates ZEB1 on the transcriptional level. (**a**) PC3, H1299, and SKOV3 cells were pre-incubated in serum free media for 24 h and then treated with or without MLN4924 for 24 h. The cell lysates were subjected to western blot analysis using the indicated antibodies. (**b**) PC3, H1299, and SKOV3 cell lines were transfected with si-C or si-N8. The cell lysates were subjected to western blot analysis using the indicated antibodies. (**c**) PC3, H1299, and SKOV3 cells were incubated with 0.125 μM MLN4924 for the indicated times. ZEB1 mRNA levels were analyzed by RT-qPCR. (**d**) PC3, H1299, and SKOV3 cells were incubated with 0.125 μM MLN4924 and 2 nM Actinomycin D for 24 h. ZEB1 mRNA levels were analyzed by RT-qPCR. Data are presented as the means ± SD (n = 3). *p < 0.05; **p < 0.01; ***p < 0.001; *ns* not significant.

Since the EMT is a hallmark of metastasis, we investigated various EMT markers following treatment with MLN4924 and NEDD8-targeting siRNA (si-N8) in each cell line. While PC3 showed an adverse result in E-cadherin and N-cadherin levels with MLN4924 treatment, the other two cell lines showed no significant change (Fig. 2a). Although there was an induction in Slug expression levels for MLN4924 treated SKOV3 cells, the other two cell lines showed either weak or no expression (Fig. 2a, Supplementary Figure S4). Moreover, Vimentin showed no significant change in both MLN4924 treated and si-N8 transfected cancer cell lines (Fig. 2a,b). ZEB1 expression levels, however, were dramatically induced in all three cancer cell lines under the condition of neddylation blockade (Fig. 2a,b). As ZEB1 protein expression consistently increased, we further examined whether ZEB1 gene expression is also regulated by neddylation. Interestingly, ZEB1 mRNA levels showed a significant increase for the given time periods (12 and 24 h) in all three cancer cell lines (Fig. 2c). Given that neddylation may be involved in regulating both transcription and translation of ZEB1, we further investigated the role of neddylation blockade in ZEB1 expression using Actinomycin D. As a result, induction of ZEB1 mRNA level by MLN4924 was inhibited when treated with Actinomycin D (Fig. 2d). In addition, treatment of MLN4924 showed no change in protein stability when co-treated with CHX (Supplementary Figure S5). Therefore, these results indicate that neddylation regulates ZEB1 mainly on the transcription level.

### Blockade of neddylation induces ZEB1-driven cancer cell migration

**Figure 3:**
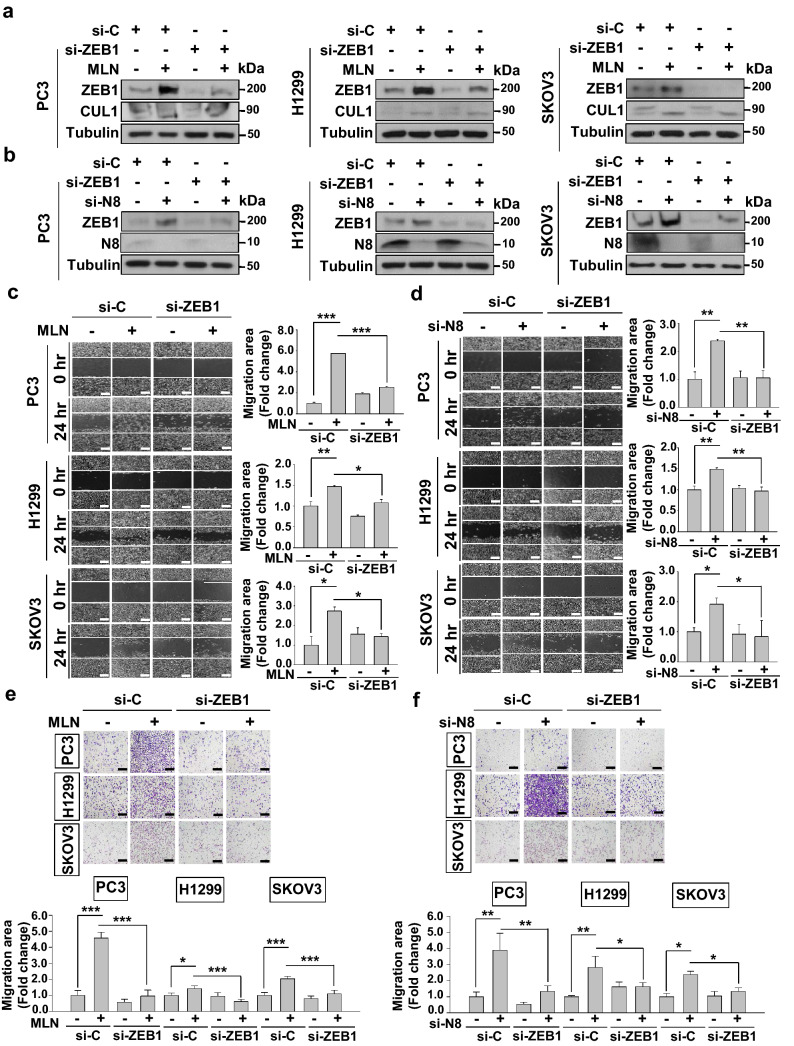
ZEB1 knockdown attenuates the cell migration-promoting effect of neddylation blockade. (**a**) PC3, H1299, and SKOV3 cells were transfected with si-ZEB1 and/or treated with MLN4924. The cell lysates were subjected to western blot analysis using the indicated antibodies. (**b**) PC3, H1299, and SKOV3 cells were transfected with si-ZEB1 and/or si-N8. Cell lysates were subjected to western blot analysis using the indicated antibodies. (**c**) PC3, H1299 and SKOV3 cells transfected with si-ZEB1 and/or treated with MLN4924 were subjected to a wound healing assay in serum-free media for 24 h. (**d**) PC3, H1299, and SKOV3 cells were transfected with si-ZEB1 and/or si-N8 and subjected to a wound healing assay in serum-free media for 24 h. Scale bar: 200 μm. Empty areas were quantified using ImageJ. Data are presented as the means ± SD (n = 3). (**e**) PC3, H1299, and SKOV3 cells transfected with si-ZEB1 and/or treated with MLN4924 were subjected to a Transwell migration assay. (**f)** PC3, H1299, and SKOV3 cells co-transfected with or without si-ZEB1 and si-N8 were subjected to a Transwell migration assay. The numbers of cells in four randomly chosen fields were counted. *p < 0.05; **p < 0.01; ***p < 0.001.

Based on the above results, we hypothesized that neddylation blockade stimulates ZEB1-driven cancer cell migration. We performed wound healing and Transwell assays in PC3, H1299 and SKOV3 cell lines using MLN4924 or si-NEDD8 with ZEB1-targeting siRNA (si-ZEB1). The results showed that the induction of cell migration by MLN4924 treatment was attenuated with si-ZEB1 (Fig. 3a,c,e). This was further confirmed using si-NEDD8, which resulted in a similar trend of migration attenuation (Fig. 3b,d,f). Thus, these results indicate that cell migration is dependent on ZEB1 when neddylation is blocked.

### ZEB1 is induced through the activation of the PI3K/Akt/mTOR pathway by neddylation blockade.

**Figure 4:**
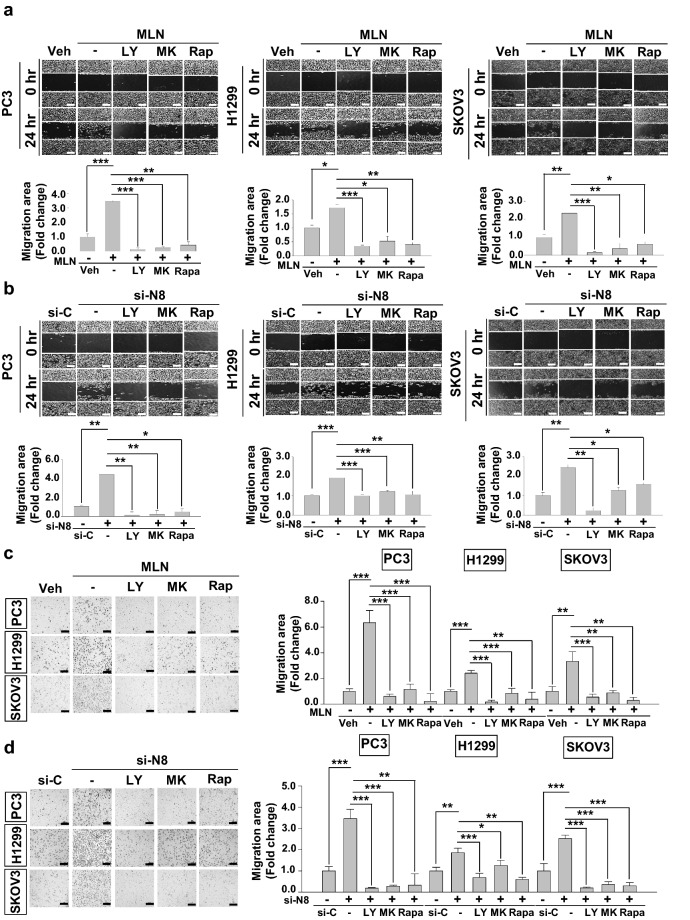

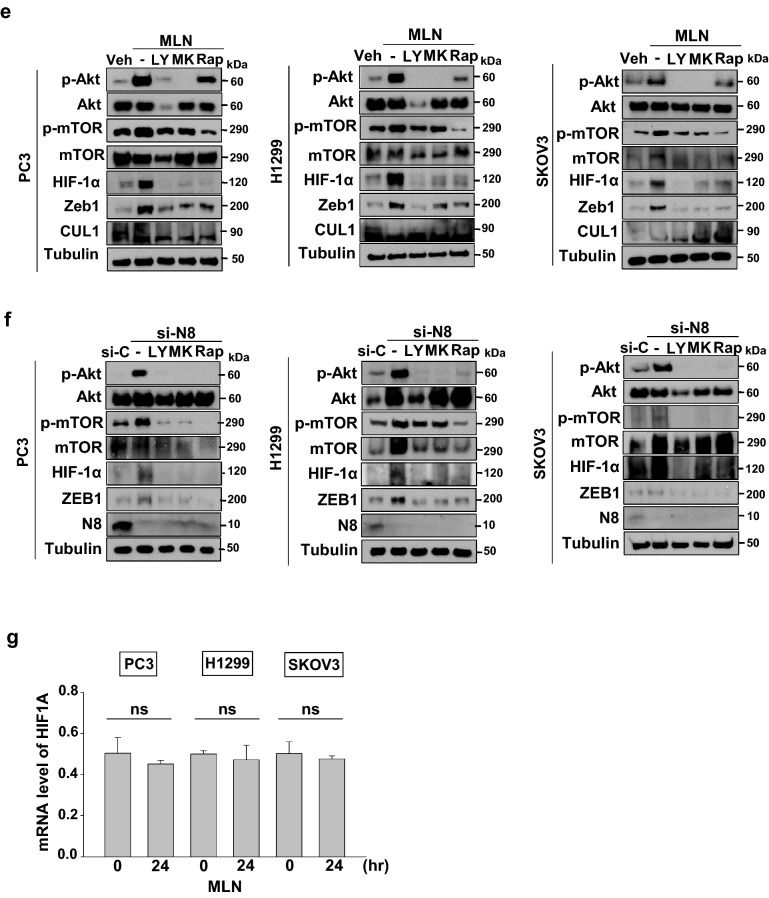
Impairment in neddylation promotes cell migration through the PI3K-AKT-mTOR pathway. (**a**) PC3, H1299, SKOV3 cells treated with MLN4924 were subjected to wound healing assay in serum-free media in the presence of 50 μM LY294002, 1 μM MK2206 or 1 μM Rapamycin for 24 h. (**b**) PC3, H1299, SKOV3 cells transfected with si-N8 were subjected to wound healing assay in serum-free media in the presence of the indicated inhibitors. Empty areas were quantified using ImageJ software (scale bar: 200 μm). (**c**) PC3, H1299, SKOV3 cells treated with MLN4924 were subjected to Transwell migration assay in the presence of the indicated inhibitors. (**d**) PC3, H1299, SKOV3 cells transfected with si-N8 were subjected to Transwell migration assay in the presence of the indicated inhibitors. The numbers of cells in four randomly chosen fields were counted. **(e)** Lysates of PC3, H1299, and SKOV3 cells treated with MLN4924 and indicated inhibitors were subjected to Western blotting with the indicated antibodies. **(f)** Lysates of PC3, H1299, and SKOV3 cells transfected with si-N8 and indicated inhibitors were subjected to Western blotting with the indicated antibodies. **(g)** PC3, H1299, and SKOV3 cells were incubated with 0.125 μM MLN4924 for the indicated times. HIF-1α mRNA levels were analyzed by RT-qPCR. Each bar represents the mean ± SD (n = 3). *p < 0.05; **p < 0.01; ***p < 0.001; *ns* not significant.

Given that neddylation blockade was shown to influence the PI3K/Akt/mTOR pathway in our previous report, we investigated the possibility of ZEB1 regulation via activation of the PI3K/Akt/mTOR pathway. We conducted both wound healing assay and transwell assay by co-treating with MLN4924 and inhibitors of PI3K/Akt/mTOR in all three cancer cell lines (Fig. 4a,c). The effect on migration brought on by the neddylation blockade was attenuated when the cells were co-treated with inhibitors. Protein levels of p-AKT and p-mTOR showed increase in treatment of MLN4924, while showing a decrease with other additional respective inhibitors. Then, we confirmed expression levels of ZEB1, which also showed attenuation in the presence of inhibitors even when neddylation was impaired (Fig. 4e). We further confirmed both the migration effects and expression levels using si-NEDD8 and treating with the same inhibitors in all three cancer cell lines (Fig. 4b,d,f). For additional confirmation, we also performed wound healing assay with the treatment of inhibitors alone for a useful comparison to the combination treatment of both MLN4924 and si-NEDD8 (Supplementary Figures S6, S7). Since HIF-1α is known to be regulated specifically at the protein level by the activation of PI3K/Akt/mTOR pathway^13,15,16^ and ZEB1 is known to be regulated by HIF-1α^10^, we confirmed the expression level of HIF-1α as well. We found that HIF-1α expression showed a trend towards attenuation similar to that of ZEB1 (Fig. 4e,f). In addition, HIF-1α mRNA expression did not change following treatment with MLN4924 in PC3, H1299 and SKOV3 cells (Fig. 4g). Hence, the following results suggest neddylation blockade induces ZEB1 through the activation of the PI3K/Akt/mTOR pathway dependent HIF-1α induction.

### Induction of HIF-1α by neddylation blockade promotes cell migration by directly regulating ZEB1

**Figure 5:**
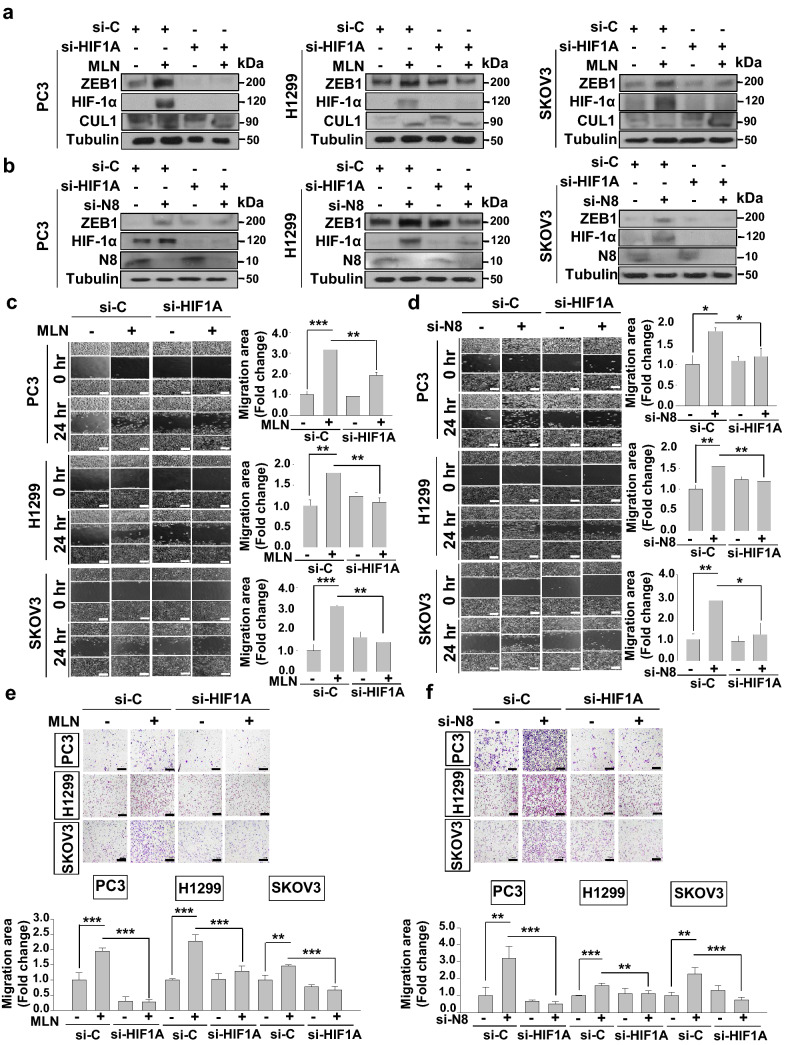
HIF-1α knockdown attenuates the cell migration-promoting effect of neddylation blockade. (**a**) PC3, H1299, and SKOV3 cells were transfected with si-HIF1A and/or treated with MLN4924. The cell lysates were subjected to western blot analysis using the indicated antibodies. (**b**) PC3, H1299, and SKOV3 cells were transfected with si-HIF1A and/or si-N8. Cell lysates were subjected to western blot analysis using the indicated antibodies. (**c**) PC3, H1299 and SKOV3 cells transfected with si-HIF1A and/or treated with MLN4924 were subjected to a wound healing assay in serum-free media for 24 h. (**d**) PC3, H1299, and SKOV3 cells were transfected with si-HIF1A and/or si-N8 and subjected to a wound healing assay in serum-free media for 24 h. Scale bar: 200 μm. Empty areas were quantified using ImageJ. Data are presented as the means ± SD (n = 3). (**e**) PC3, H1299, and SKOV3 cells transfected with si-HIF1A and/or treated with MLN4924 were subjected to a Transwell migration assay. (**f)** PC3, H1299, and SKOV3 cells co-transfected with or without si-HIF1A and si-N8 were subjected to a Transwell migration assay. The numbers of cells in four randomly chosen fields were counted. *p < 0.05; **p < 0.01; ***p < 0.001.

Since ZEB1 is known to be regulated by HIF-1α and HIF-1α is also known to be neddylated^17^, we tested whether HIF-1α is responsible for the induction of ZEB1 in the presence of neddylation blockade. Transfection of HIF-1α-targeting siRNA (si-HIF1A) in all cell lines and treatment with MLN4924 or co-transfection of si-NEDD8 revealed that induction of ZEB1 during neddylation blockade is significantly dependent on HIF-1α (Fig. 5a,b). As induction of ZEB1 expression is dependent on HIF-1α expression when neddylation is blocked, we further investigated whether HIF-1α plays a role in promoting cell migration during neddylation blockade. Wound healing and Transwell assays were performed in si-HIF1A transfected PC3, H1299, SKOV3 cell lines with or without MLN4924. Knockdown of HIF-1α significantly attenuated the migration effect during neddylation blockade (Fig. 5c,e). This effect was confirmed in all three cancer cell lines by co-transfection of si-NEDD8 and si-HIF1A (Fig. 5d,f). Thus, our results represent that neddylation blockade promotes cell migration through HIF-1α by directly regulating the EMT transcription factor ZEB1.

## Discussion

To the best of our knowledge, the present study is the first to illustrate the association between neddylation and EMT transcription factor ZEB1 through induction of HIF-1α. Our results confirmed there was a significant decrease in NEDD8 mRNA in prostate cancer tissues compared to normal tissues (Fig. 1a). Since the data from lung tumor samples showed a decreasing trend in NEDD8 mRNA expression, and considering the possible error resulting from human data collection and small sample sizes, we verified the molecular mechanism involved in cell migration and neddylation in vitro through prostate (PC3), lung (H1299), ovarian (SKOV3) derived cancer cell lines. We used both the NEDD8-activating enzyme inhibitor MLN4924 and silencing of NEDD8 to confirm the increase in cell migration. Following neddylation blockade, we discovered that induction of ZEB1 was mediated by HIF-1α through the activation of the PI3K/Akt/mTOR pathway, emphasizing the role of neddylation in cancer migration.

Metastasis, or the dissemination of a pathogenic agent from a primary site to a secondary site, has been vigorously investigated through genetic variations, including modifications of oncogenic proteins. In the present study, we mainly focused on metastasis in regards to neddylation, which is similar to ubiquitination in that it targets a variety of proteins, including oncoproteins. For instance, Von Hippel-Lindau (VHL) modification by NEDD8 is significant for tumor suppression^18^. In addition, overactivated neddylation in lung adenocariconma pateients has been shown to be associated with a lower overall survival rate^19^. Thus, MLN4924 has potential as an anti-tumour reagent. However, in contrast with many reports regarding MLN4924 as an anticancer reagent inhibiting tumor progression, Zhou et al. reported increased tumor-sphere formation and stimulation of cancer cell migration when neddylation was blocked^20^. Similarly, in the previous studies we observed that neddylation blockade promoted cancer cell migration. For instance, neddylation blockade induced Src-mediated phosphorylation of caveolin-1, which has been reported to act as a tumor promoter^5^. In addition, we observed C-CBL function as an E3 ligase for c-Src neddylation, which resulted in promoting cancer cell migration through activation of the PI3K/Akt pathway^14^. These results suggest that neddylation blockade plays a significant role in modifying oncogenic proteins and downstream factors beyond the PI3K/Akt pathway. Thus, we questioned the possible involvement of EMT in the activation of the PI3K/Akt pathway when neddylation is blocked.

ZEB1 plays a critical role as one of the main transcription factors involved in the EMT. Although studies on the role of the EMT in cancer has produced conflicting results, the induction of particular transcription factors has been shown to play a significant part in metastasis and invasion by increasing cancer cell motility^21^. Among these factors, the induction of ZEB1 transcriptionally represses many epithelial marker genes, most notably E-cadherin, which is repressed when ZEB1 binds E box sequences in its promoter region^22^. Additionally, microRNA-200 family members that induce epithelial differentiation are known to be transcriptionally repressed by ZEB1 during the EMT^23^. Regarding the role of ZEB1 as a transcription factor of EMT, however, our findings suggest that induction of ZEB1 could be an independent mechanism of the EMT since only ZEB1 showed consistent induction in all three cancer cell lines (Fig. 2a,b). An EMT-independent mechanism for the biological functions of ZEB1 has also been reported in epidermal growth factor receptor (EGFR)-mutated lung cancer cells^24^. Though EGFR mutation may not be the case in our data, our results demonstrate the functional role of ZEB1 through genetic depletion and blockade of neddylation (Fig. 3).

ZEB1 is considered a main downstream target of many oncogenic pathways including the PI3K/Akt pathway^25^. PI3K/Akt/mTOR pathway plays an exclusive role in metastasis by regulating numerous oncoproteins^26^. For instance, PI3K/Akt pathway activation has been significantly linked to the increased survival of breast cancer cells and subsequent loss of the PTEN gene^27^. In addition, the PI3K/Akt/mTOR pathway has been reported to cause mutations in tuberous sclerosis complex 1/2 (TSC1 and TSC2) genes and LKB1 genes, which have been linked to increased rates of cancer^28,29^. Here, we demonstrate that activation of PI3K/Akt/mTOR pathway during neddylation blockade is responsible for ZEB1 induction (Fig. 4). Furthermore, the PI3K/Akt pathway is also known for consequently regulating HIF-1α, a transcription factor involved in regulating many target genes in which the protein products are recognized for their significant roles in cancer-related processes^30,31^. More specifically, HIF-1α expression is induced by the activation of PI3K/Akt/mTOR pathway independent from its protein stability. Activation of the PI3K/Akt pathway induces HIF-1α expression^13^, while mTORC1 induces mRNA translation of HIF-1α^16^. Such notions explain our results and indicate the specific induction of HIF-1α expression independent of the HIF-1α transcription level (Fig. 4g).

Upregulation of HIF-1α expression is a common phenomenon in metastasis or aggressive phenotypes in many cancers such as lung, prostate, breast and pancreas carcinomas^32,33^. Activated by the common cancer cell migration pathway, HIF-1α plays significant roles in EMT transcription factors. For instance, HIF-1α is reported to regulate Snail and β-catenin pathways in pulmonary fibrosis^34^. ZEB1 is reported to be a direct downstream of HIF-1α in colorectal cancer^35^. In fact, HIF-1α is known to directly bind to the HRE-3 site containing promoter of ZEB1, which has been confirmed through ChiP assay and EMSA assay^35,36^. In our findings, neddylation blockade caused induction of HIF-1α by the activation of PI3K/Akt/mTOR pathway, thereby inducing ZEB1 expression (Fig. 4e,f). In addition, Zhou et al. discovered the promotion of wound healing in an epidermal growth factor-induced skin mouse model, which suggests that neddylation blockade is involved in enhancing cell migration^20^. In fact, findings regarding MLN4924 as an anti-tumor reagent have been reported in previous studies^37,38^ and MLN4924 has been assessed in phase I and II clinical trials and undergoing other trials to evaluate its use as an anti-tumor reagent^39,40^. Thus, as we have discovered unexpected outcomes concerning the induction of cancer cell migration, developing MLN4924 as an anticancer drug should be approached in a more careful manner.

## Materials and methods

### Informatics analysis

Publically available cancer microarray data from NCBI Gene Expression Omnibus (www.ncbi.nlm.nih.gov/gds) was analyzed to compare NEDD8 mRNA expression between normal and cancer tissues. Datasets for lung cancer (GSE108055), prostate cancer (GSE32571) and ovarian cancer (GSE29450) were selected, and the NEDD8 mRNA levels were evaluated between the groups using Mann–Whitney U test.

### Cell culture and tranfection

PC3 (human prostate cancer), H1299 (human non-small cell lung cancer), and SKOV3(human ovarian cancer) cell lines were obtained from the Korean Cell Line Bank (Seoul, Korea). PC3, H1299 were cultured in Dulbecco’s modified Eagle’s medium (DMEM) and SKOV3 was cultured in RPMI1640, supplemented with 10% heat-inactivated FBS. All cells were grown in a humidified atmosphere constituting 5% CO_2_ at 37 °C. ZEB1, NEDD8, HIF1A, and control siRNAs were purchased from M. Biotech (Hanam-si, Gyeonggi-do, South Korea). siRNA sequences are summarized in Supplementary Table S2. The siRNAs were transfected into PC3, H1299, and SKOV3 cells using Lipofectamine RNAiMAX (Invitrogen). The cells were incubated for 2 days and passaged for further experiments.

### Antibodies and chemicals

Primary antibodies used in this study are summarized in Supplementary Table S1. Anti-HIF-1α previously made were used as described^41^. MLN4924 was synthesized, as described^11^. LY294002 (L9908) (a PI3K inhibitor), Rapamycin (mTOR inhibitor) and MK-2206 (s1078) (an AKT inhibitor), (Selleck Chemicals, Houston, TX, USA) were purchased from the indicated companies. Actinomycin D (A1410) was purchased from Sigma Aldrich. CHX (C1988) was purchased from Sigma Aldrich.

### Wound healing assay

Cells transfected with appropriate vectors were cultured in a 5% CO_2_ incubator at 37 °C until confluence (80–100%). Cells were pre-incubated with serum-free media to reduce proliferation rate. The cells were then scratched with an autoclaved 200 μl pipette tip, washed twice with serum-free media to remove debris, and incubated in serum-free media for 24 h. The wounded areas were scrutinized under three randomly selected fields at the lesion border using an inverted microscope. The area of migration was measured using ImageJ software (National Institutes of Health, Bethesda, MD, USA).

### Transwell migration assay

Transwell migration assay was performed as previously described^14^. Each cells were cultured in Boyden chambers according to the manufacturer’s protocol. Cells were seeded in the 0.5 mg/mL collagen coated upper chamber insert, and the bottom chambers were filled with 500 μL of complete medium containing 10% FBS. After 24 h of incubation, the non-invading cells were removed from the membrane surface of the upper well using a cotton swab and the migrated cells were fixed with MeOH and stained using a solution containing 0.1% crystal violet in 2% methanol. Images were acquired using an inverted microscope, and the number of cells that migrated to four independent areas per filter was counted using ImageJ software.

### Immunoblotting

Immunoblot assays were performed as previously described^14^. Cell lysates were separated on SDS/polyacrylamide gels and transferred to Immobilon-P membranes (Millipore, Bedford, MA, USA). Membranes were blocked with 1% skim milk and bovine serum albumin (BSA) in Tris-buffered saline containing 0.1% Tween 20 (TTBS) for 1 h, and incubated overnight with primary antibody (dilution, 1:1000) in the blocking solution or TTBS with 1% BSA, respectively. Membranes were further incubated with a horseradish peroxidase-conjugated secondary antibody for 1 h and visualized using the ECL Plus kit (Thermo Fisher Scientific, Waltham, MA, USA).

### RNA isolation and RT-qPCR

RNA isolation and RT-qPCR were performed as previously described^14^. Total RNA was isolated using TRIzol reagent (Invitrogen, Carlsbad, CA, USA). cDNA was synthesized using an EasyScript cDNA Synthesis Kit (Applied Biological Materials Inc., Richmond, Canada) while amplified with EvaGreen qPCR master mix reagent (Applied Biological Materials) using a StepOne Real-time PCR System (Applied Biosystems, Foster City, CA, USA); 18 s ribosomal RNA was used as an internal control. Primers targeting ZEB1 (FOR: 5′-GCCAATAAGCAAACGATTCTG-3′ and REV: 5′-TTTGGCTGGATCACTTTCAAG-3′) and HIF-1α (FOR: 5′-TGCAGAATGCTCAGAGAAAGCGAA-3′ and REV: 5′-GCTGCATGATCGTCTGGCTGCT-3′) were used in the experiment.

### Statistical analysis

All experiments were repeated on three independent occasions and analyzed using Microsoft Excel 2013 (Microsoft Corp., Redmond, WA, USA) or GraphPad Prism 5 (GraphPad Inc., La Jolla, CA, USA) software. Results are expressed as means and standard deviation. Student’s *t* test was used for general statistical analyses and Mann–Whitney U test for comparing protein levels^14^. P values less than 0.05 were considered to be statistically significant. Protein expression correlations were analyzed using Spearman’s p statistics.

## Supplementary information

Supplementary figures

Supplementary tables
