# Supplementary material for: Neddylation blockade induces HIF-1α driven cancer cell migration via upregulation of ZEB1
Source: Sci Rep. 2020 Oct 23;10:18210. doi: 10.1038/s41598-020-75286-0 (PMC7585416; doi:10.1038/s41598-020-75286-0)

Supplementary Figure S1.

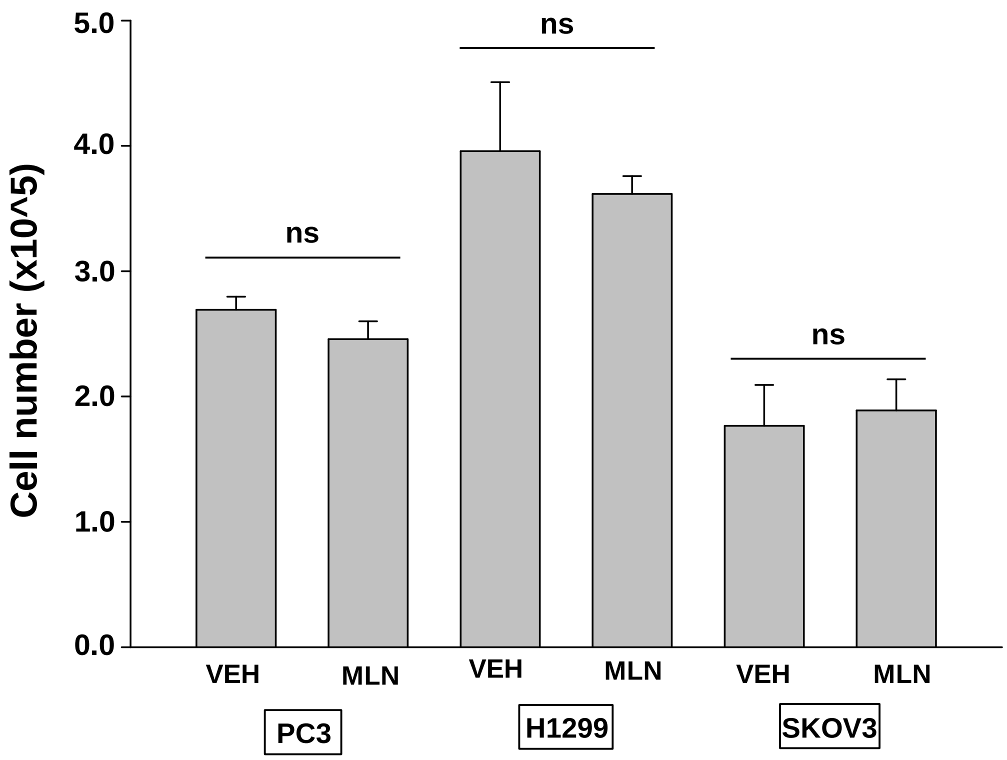

Figure S1. **Neddylation blockade does not affect cell numbers.** Quantified presentation of H1299, PC3, and SKOV3 cell numbers. All cells were pre-incubated in serum free media for 24 h and then treated with or without MLN4924 for 24 h. Cell counting was performed using a hemocytometer. Bars are the means ± SD (n = 3).

Supplementary Figure S2.

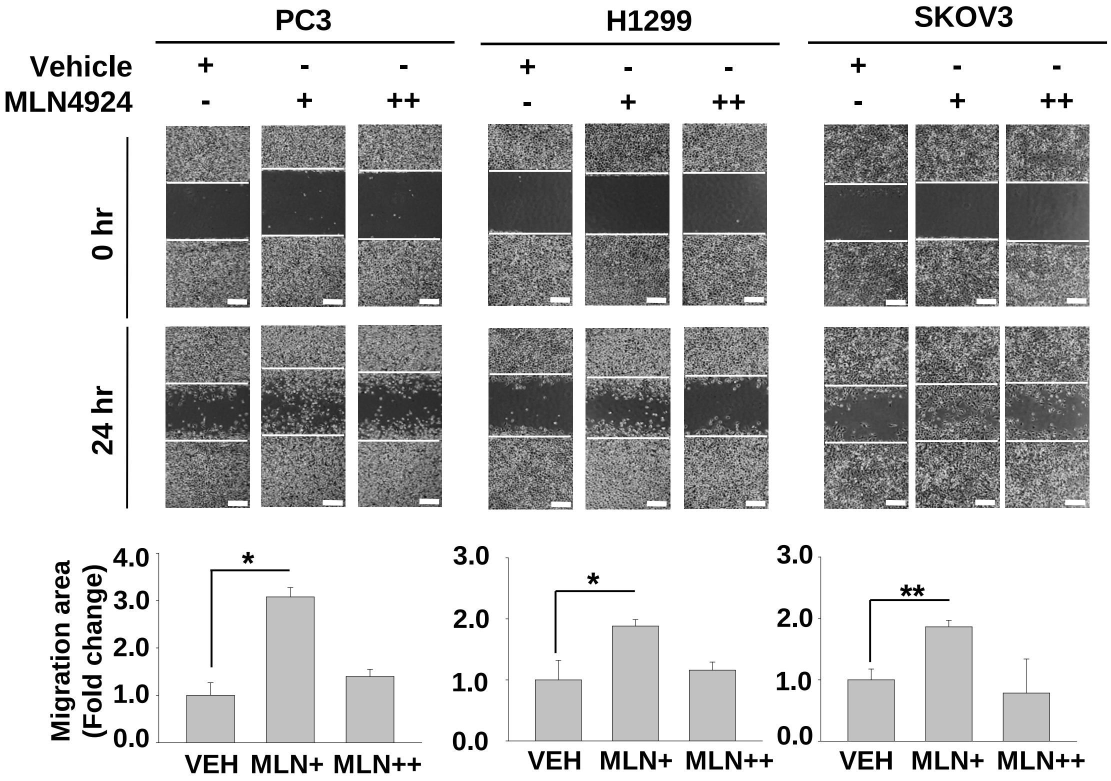

Figure S2. **Specific concentration of MLN4924 further promotes cell migration.** PC3, H1299, and SKOV3 cells were incubated in serum-free media for 24h, followed by treatment with either MLN4924 0.125μM (+) or 0.25μM (++) for 24h for wound healing assay. Scale bar: 200μM. Empty areas were quantified using ImageJ. Data are presented as the means ±SD ($n = 3$). *, $p<0.05$; **, $p<0.01$

Supplementary Figure S3.

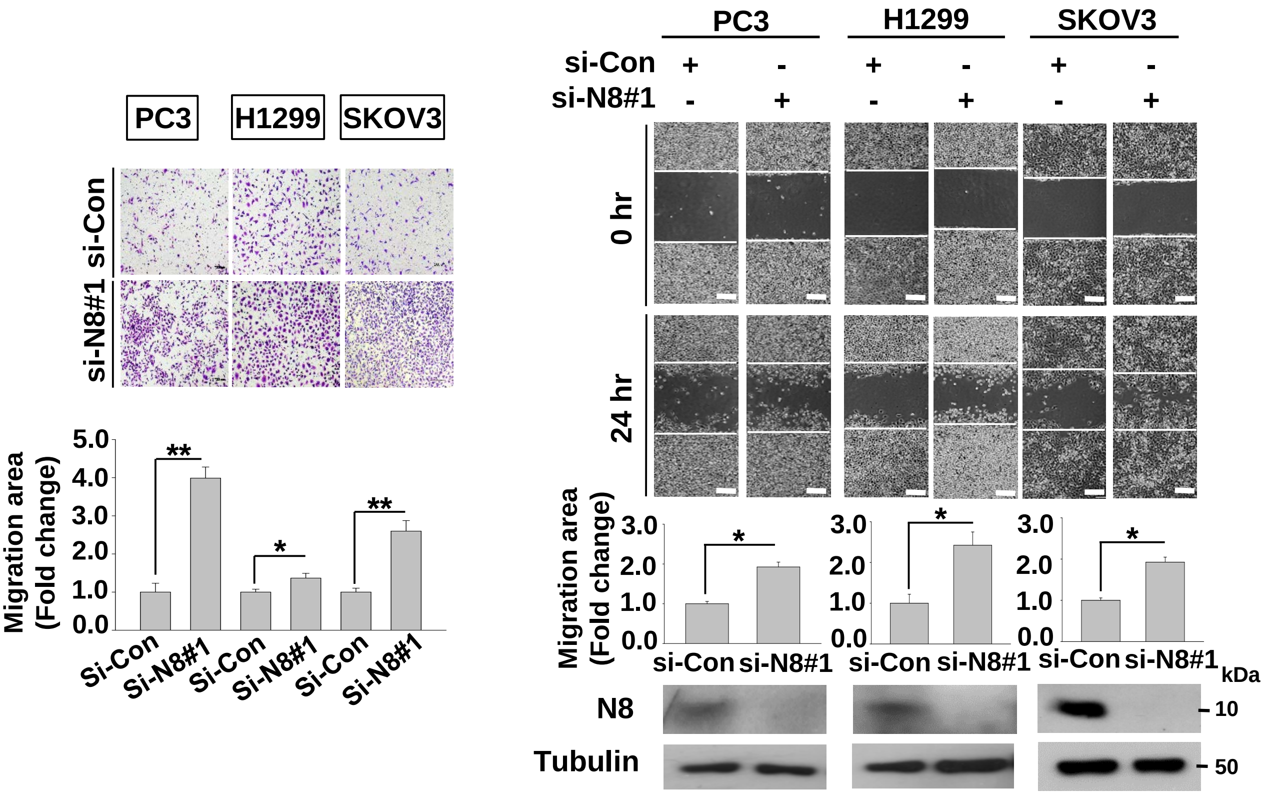

Figure S3. **Additional siRNA of NEDD8 promotes cancer cell migration.** PC3, H1299, and SKOV3 cells were transfected with si-Control of si-NEDD8#1 for wound healing assay. The cell lysates were subjected to western blot analysis using the indicated antibodies. Scale bar: 200µM. Empty areas were quantified using ImageJ. Data are presented as the means ±SD (*n* = 3). *, p<0.05; **, p<0.01

Supplementary Figure S4.

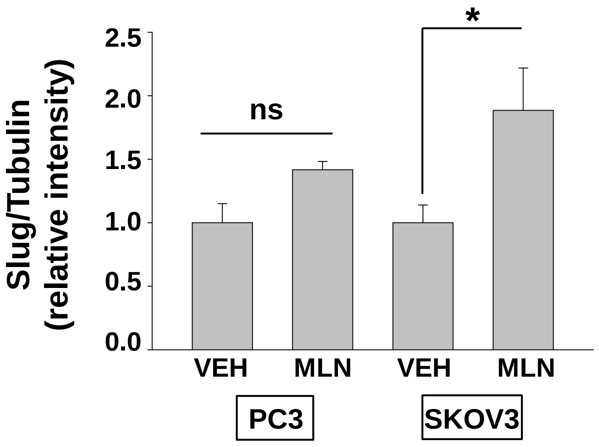

Figure S4. **Quantification of Slug protein.** PC3 and SKOV3 cells were treated with MLN4924 for 24 h. The cell lysates were subjected to western blot analysis. Band intensities (mean ±SD ($n = 3$) were analyzed using ImageJ and plotted. *, $p<0.05$, ns=not significant

Supplementary Figure S5.

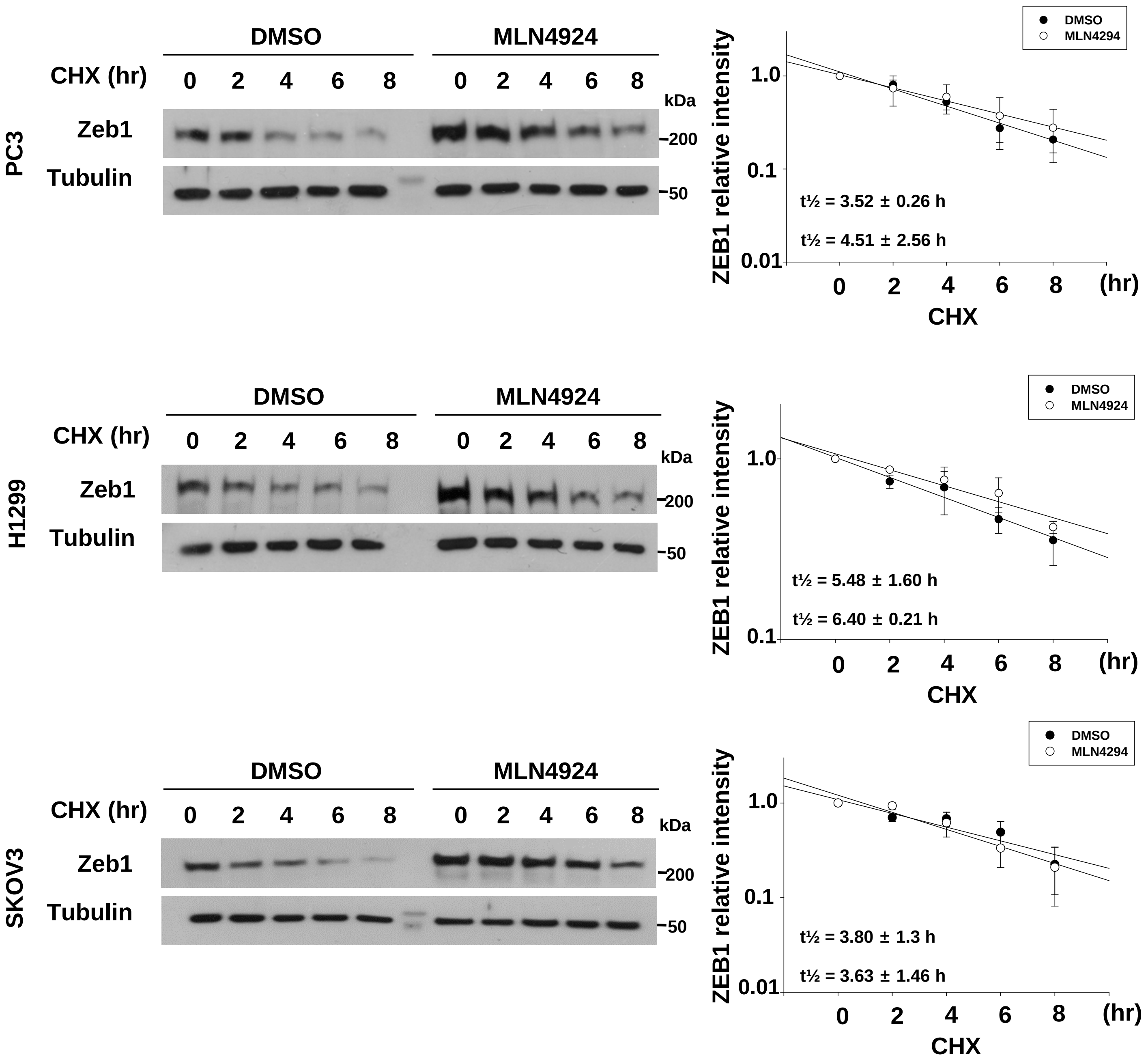

Figure S5. **Neddylation blockade does not stabilize ZEB1.** PC3, H1299, and SKOV3 cells were treated with MLN4924 for 24 h in serum-free media. Then the cells were incubated with 100μM cycloheximide (CHX) for the indicated times. Cell lysates were subjected to western blot analysis using ZEB1 antibody. Band intensities (mean ±SD ($n = 3$) were analyzed using ImageJ and plotted.

Supplementary Figure S6.

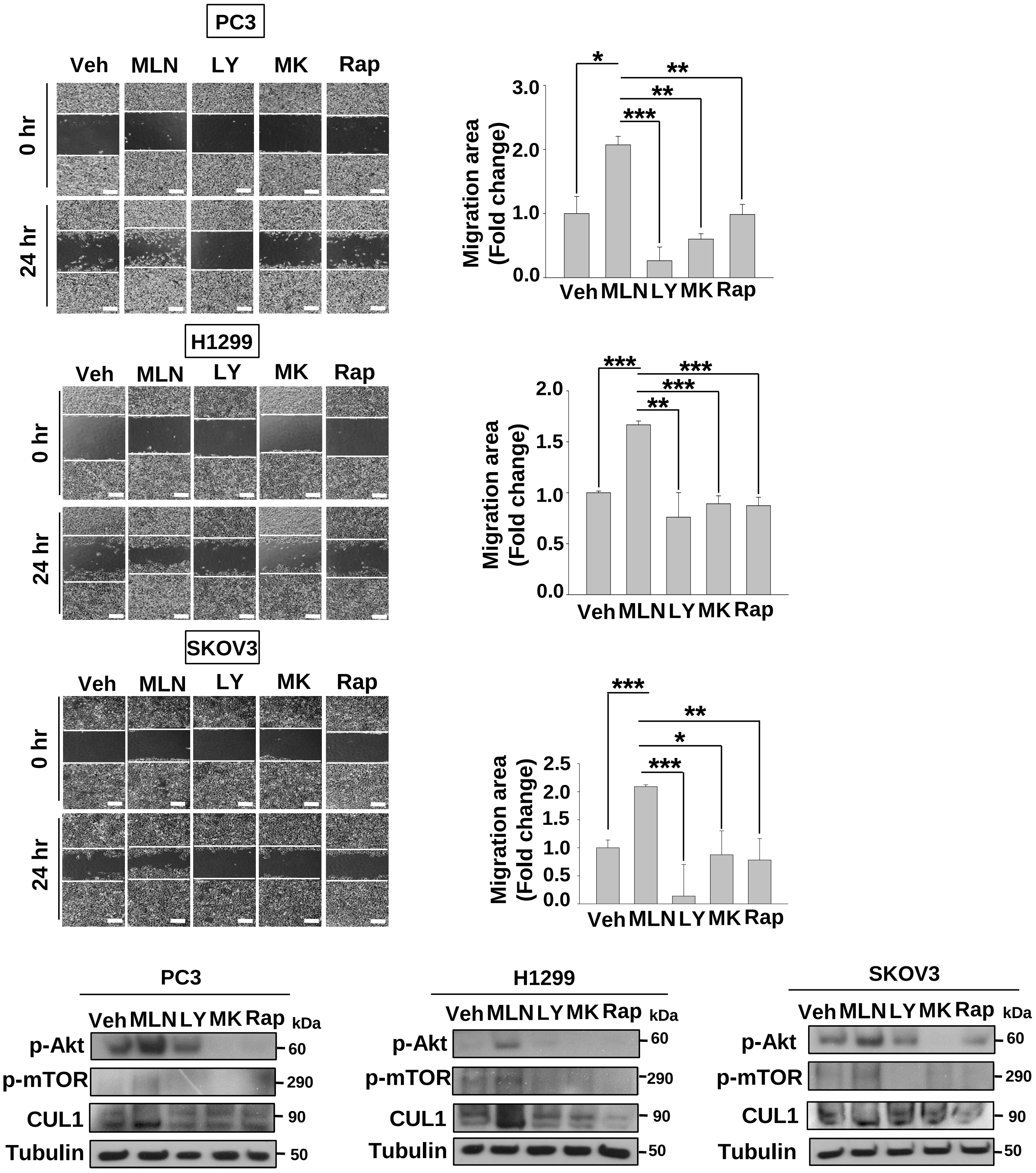

Figure S6. **PI3K/Akt/mTOR inhibitors alone results in similar migration effect with MLN4924 combined**. PC3, H1299, and SKOV3 cells were treated with MLN4924 and indicated inhibitors alone for 24 h. The cell lysates were subjected to western blot analysis. Scale bar: 200μM. Empty areas were quantified using ImageJ. Data are presented as the means ±SD ($n = 3$). *, p<0.05; **, p<0.01; ***, p<0.001

Supplementary Figure S7.

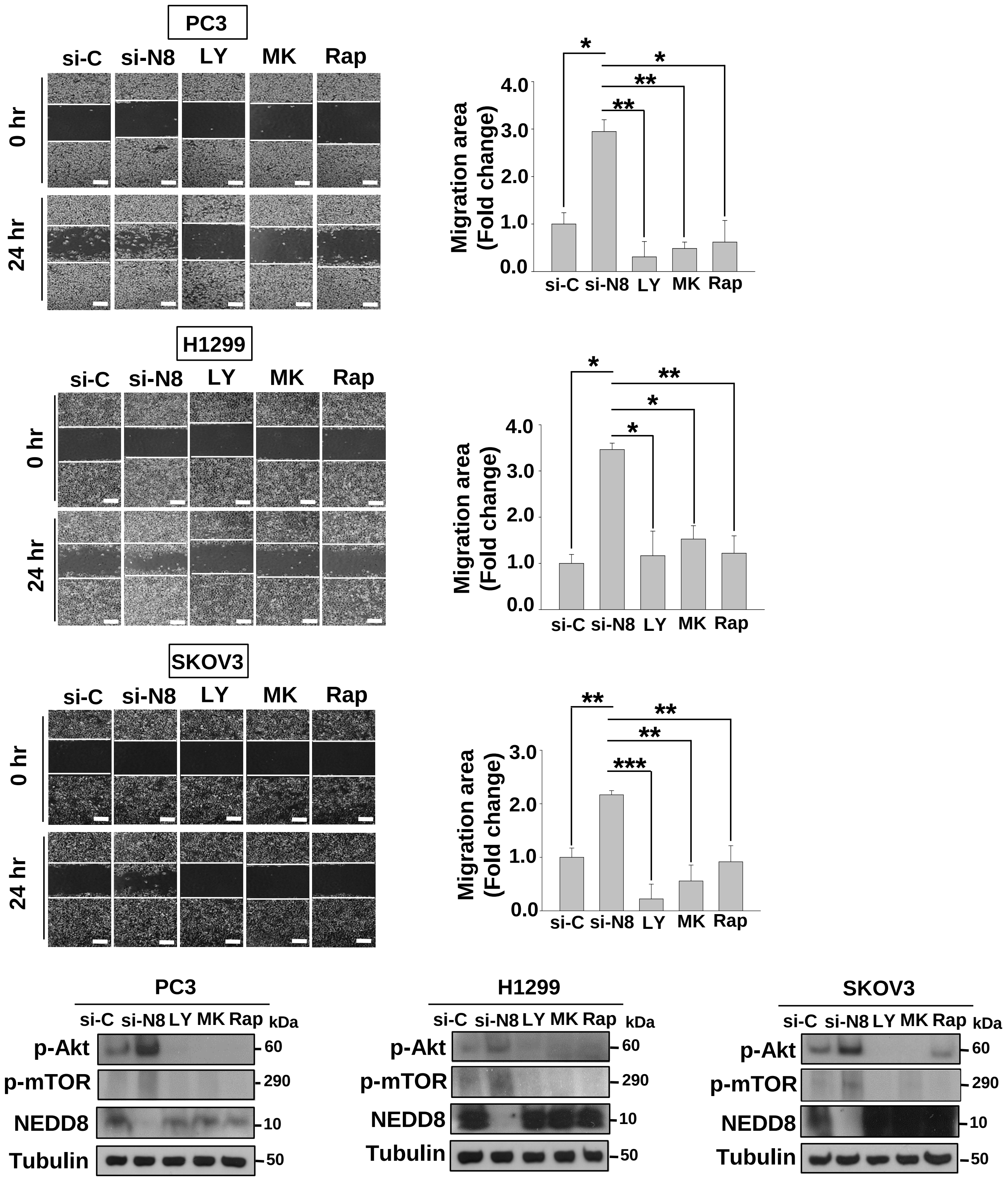

Figure S7. **PI3K/Akt/mTOR inhibitors alone results in similar migration effect with si-N8 co-treated**. PC3, H1299, and SKOV3 cells were transfected with si-N8 and indicated inhibitors alone for 24 h. The cell lysates were subjected to western blot analysis. Scale bar: 200μM. Empty areas were quantified using ImageJ. Data are presented as the means ±SD ($n = 3$). *, p<0.05; **, p<0.01; ***, p<0.001

Supplementary Figure S8.

Figure 1b

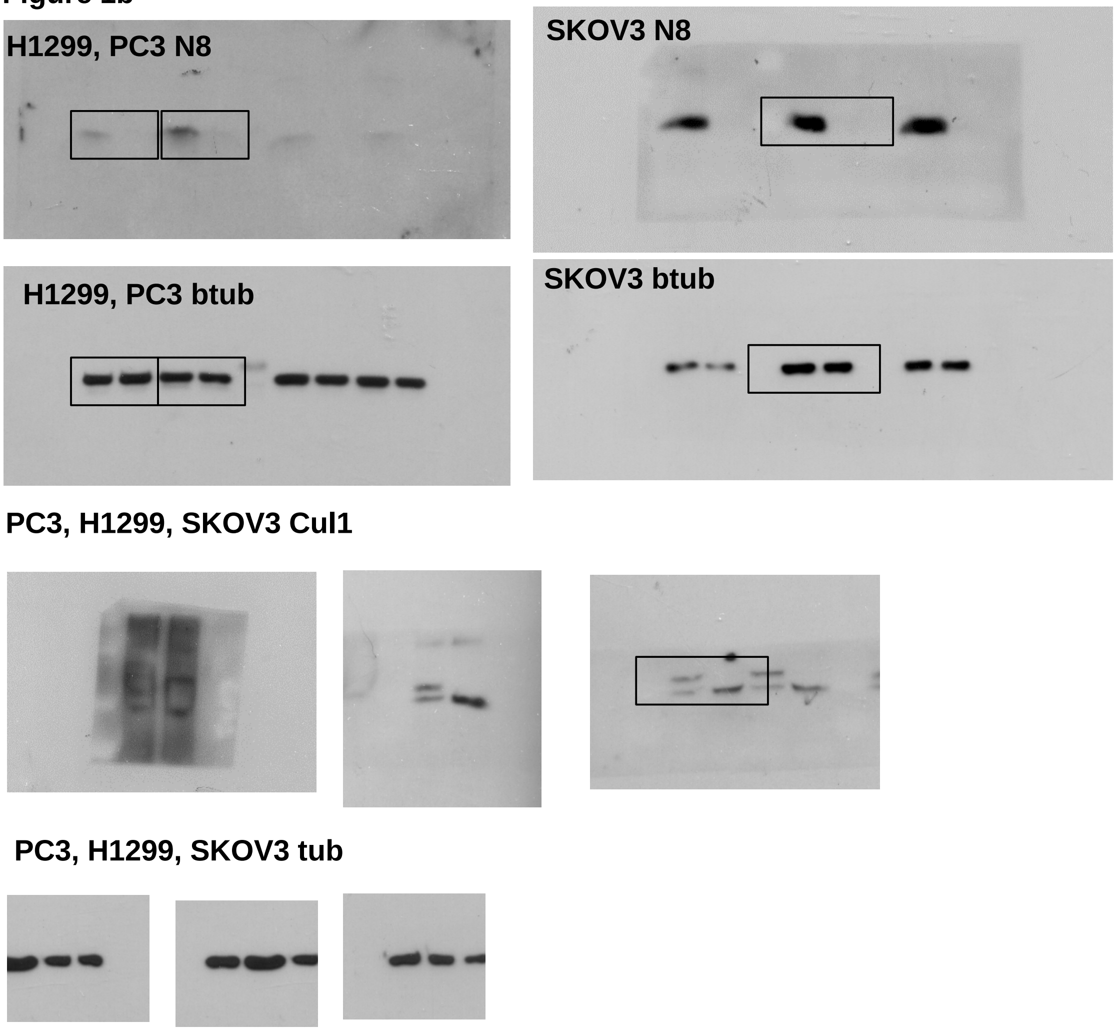

Supplementary Figure S8.

Figure 2a, 2b

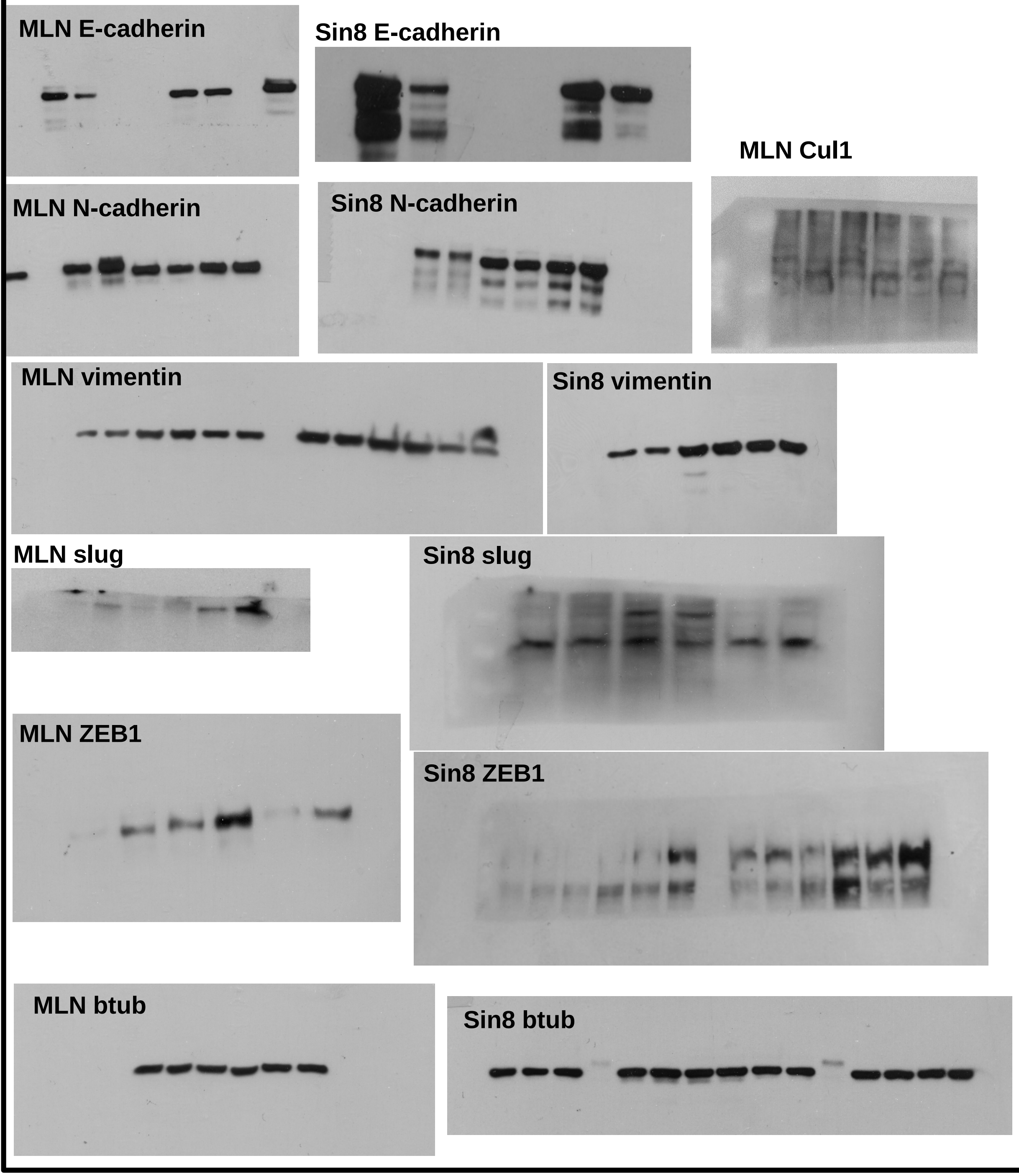

Supplementary Figure S8.

Figure 3a

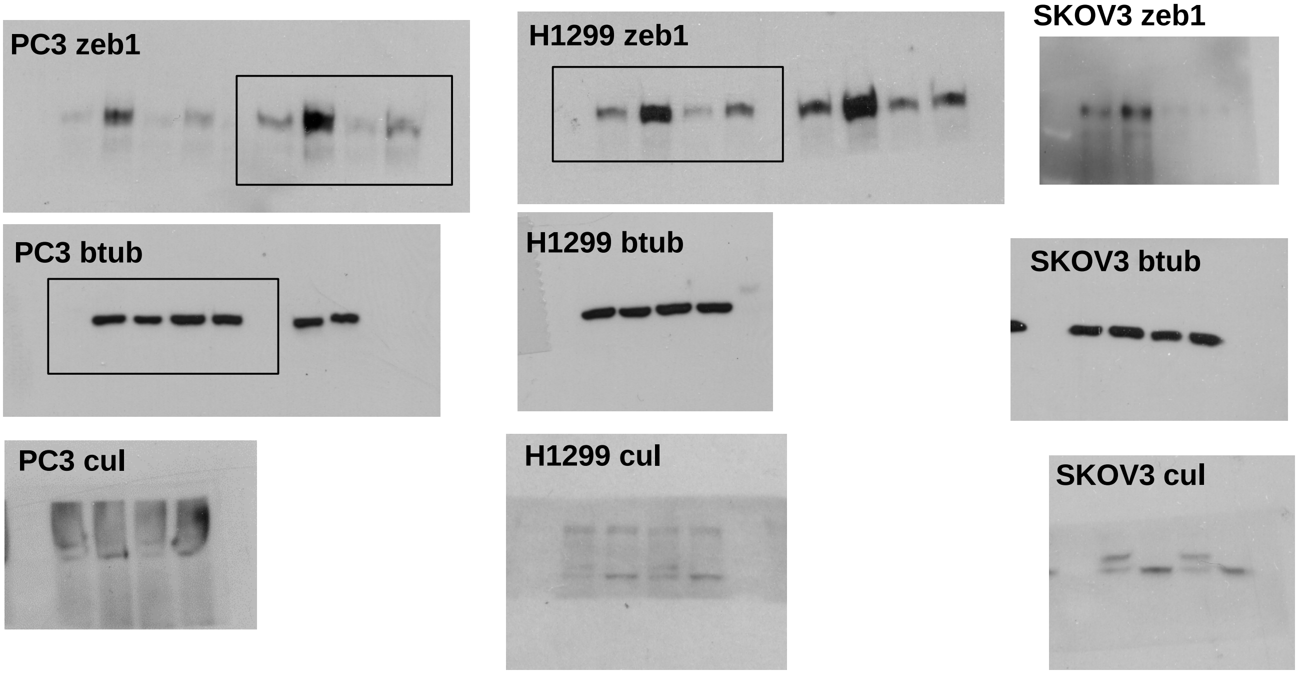

Figure 3b

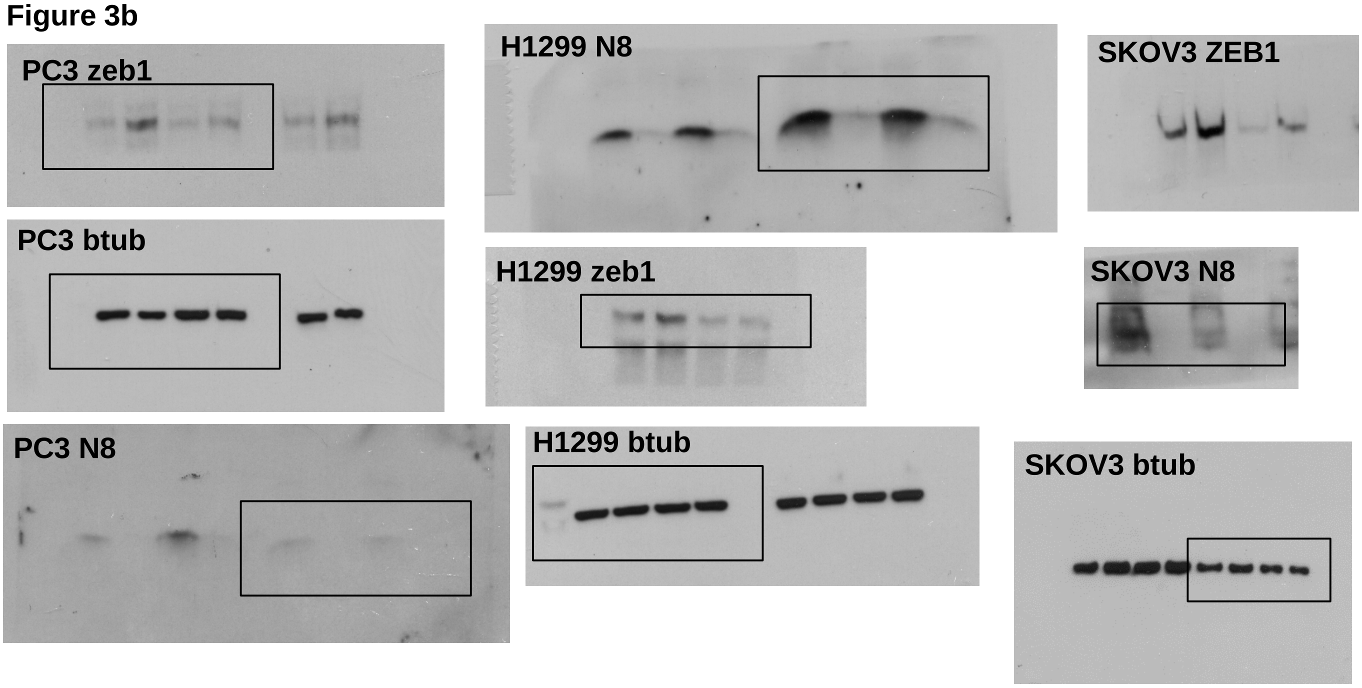

Supplementary Figure S8.

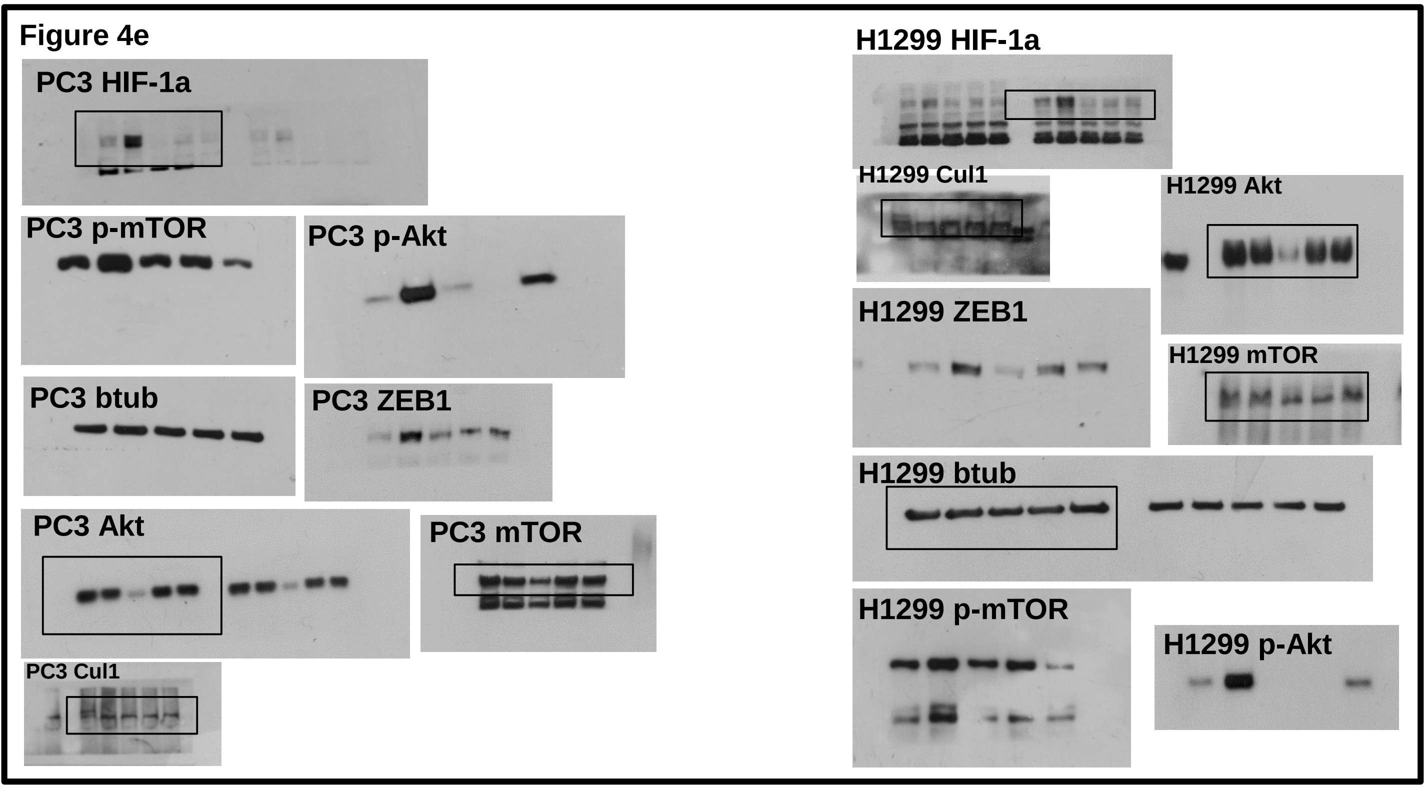

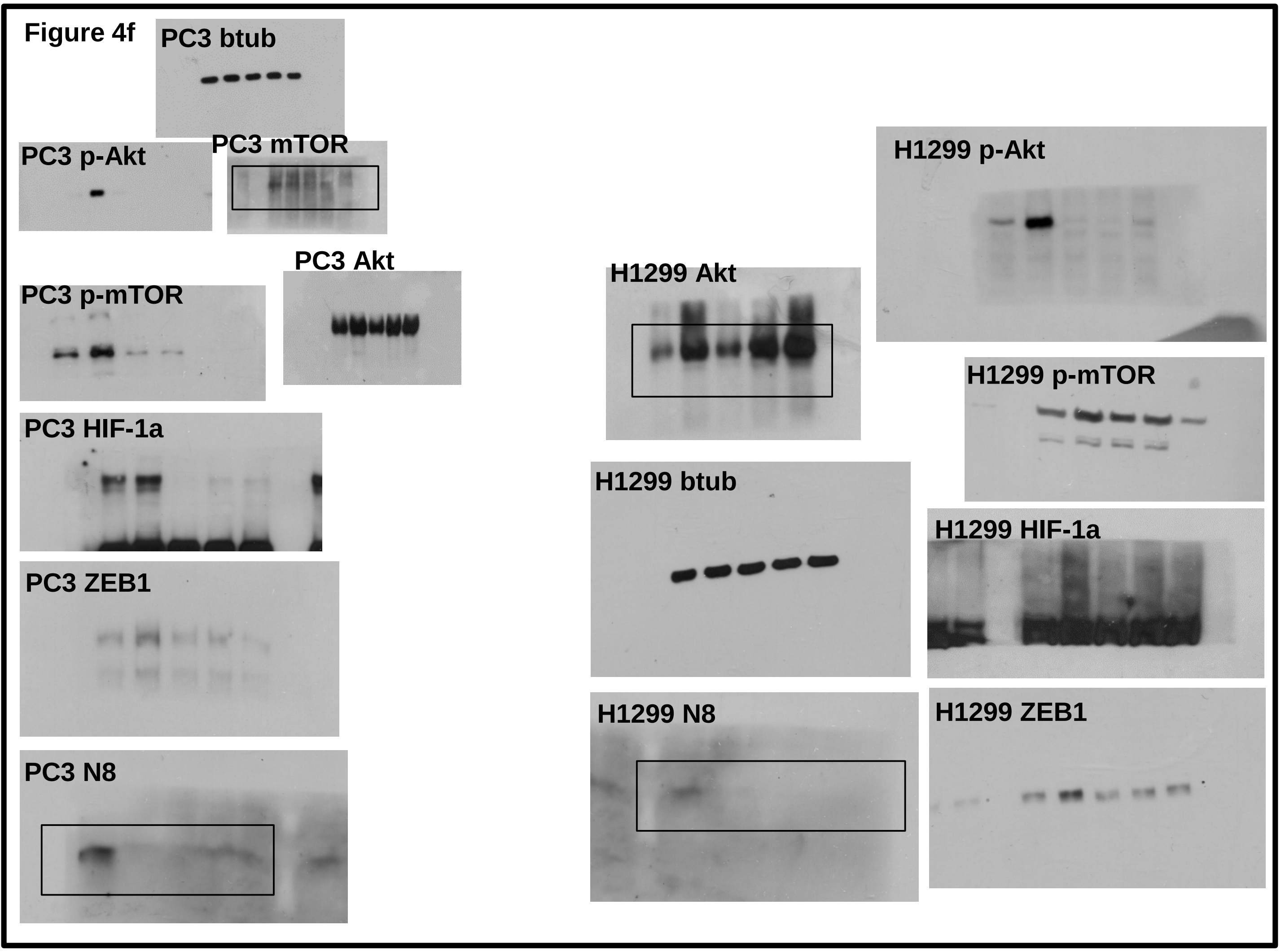

Supplementary Figure S8.

Figure 4e, f

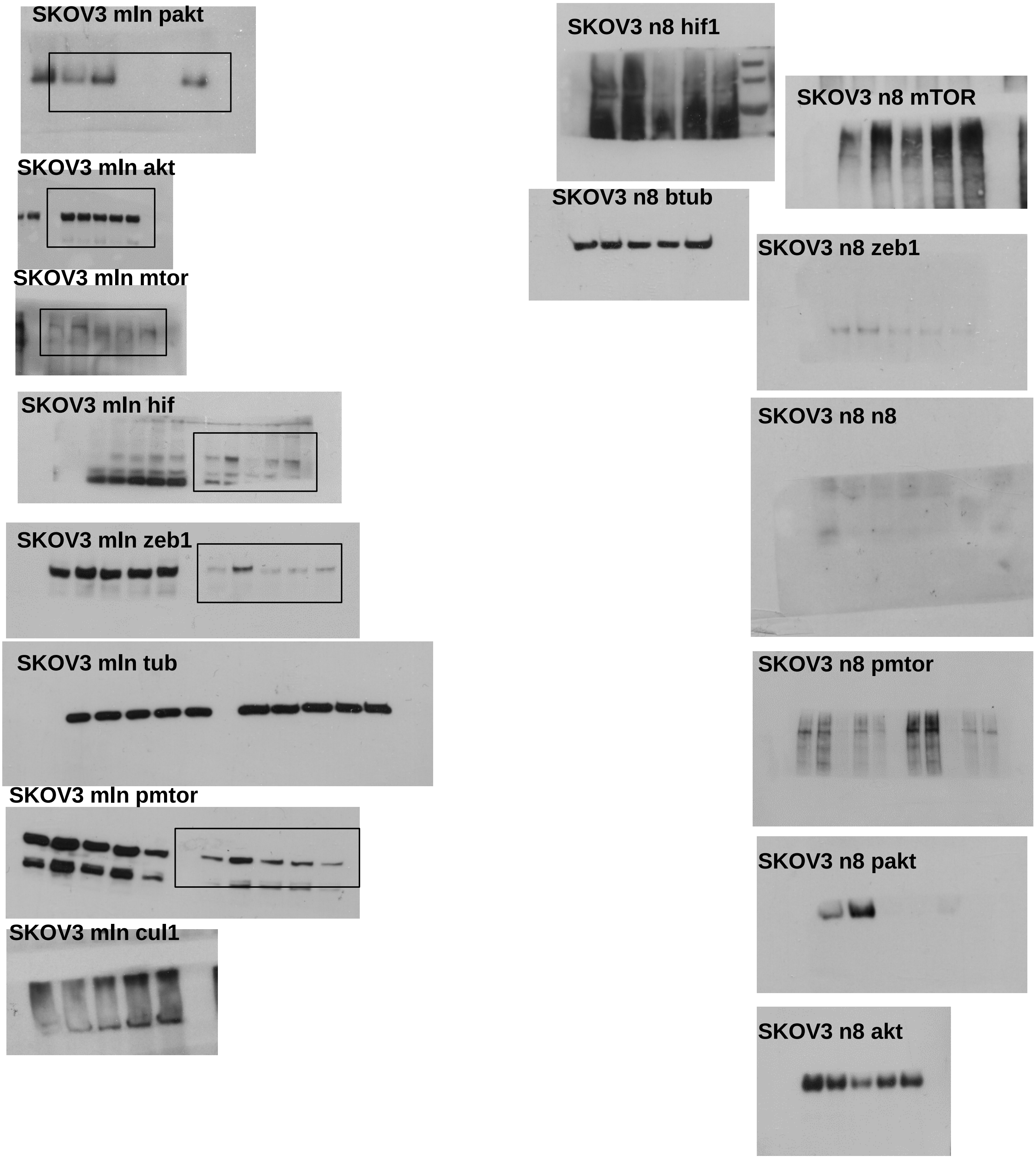

Supplementary Figure S8.

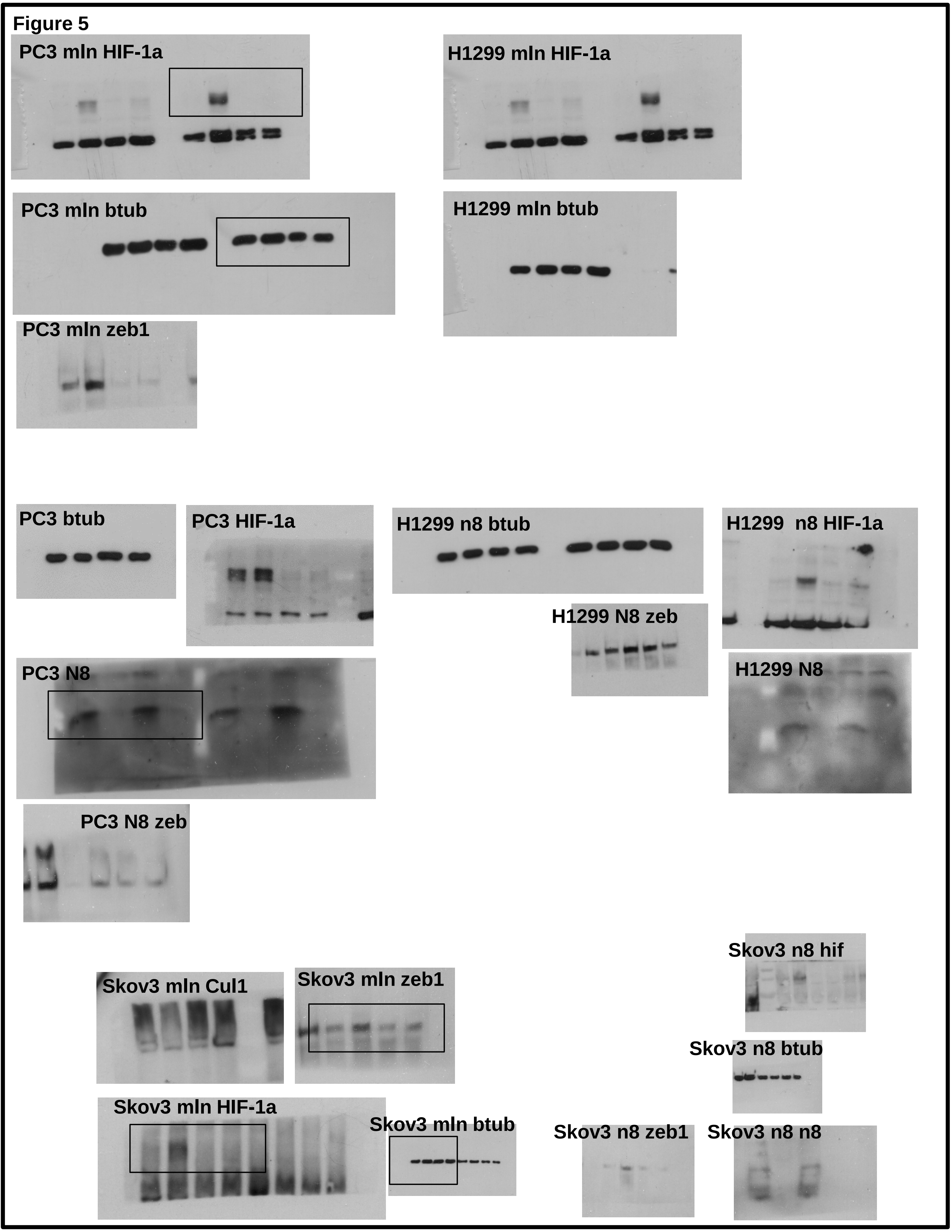

Supplementary Figure S8.

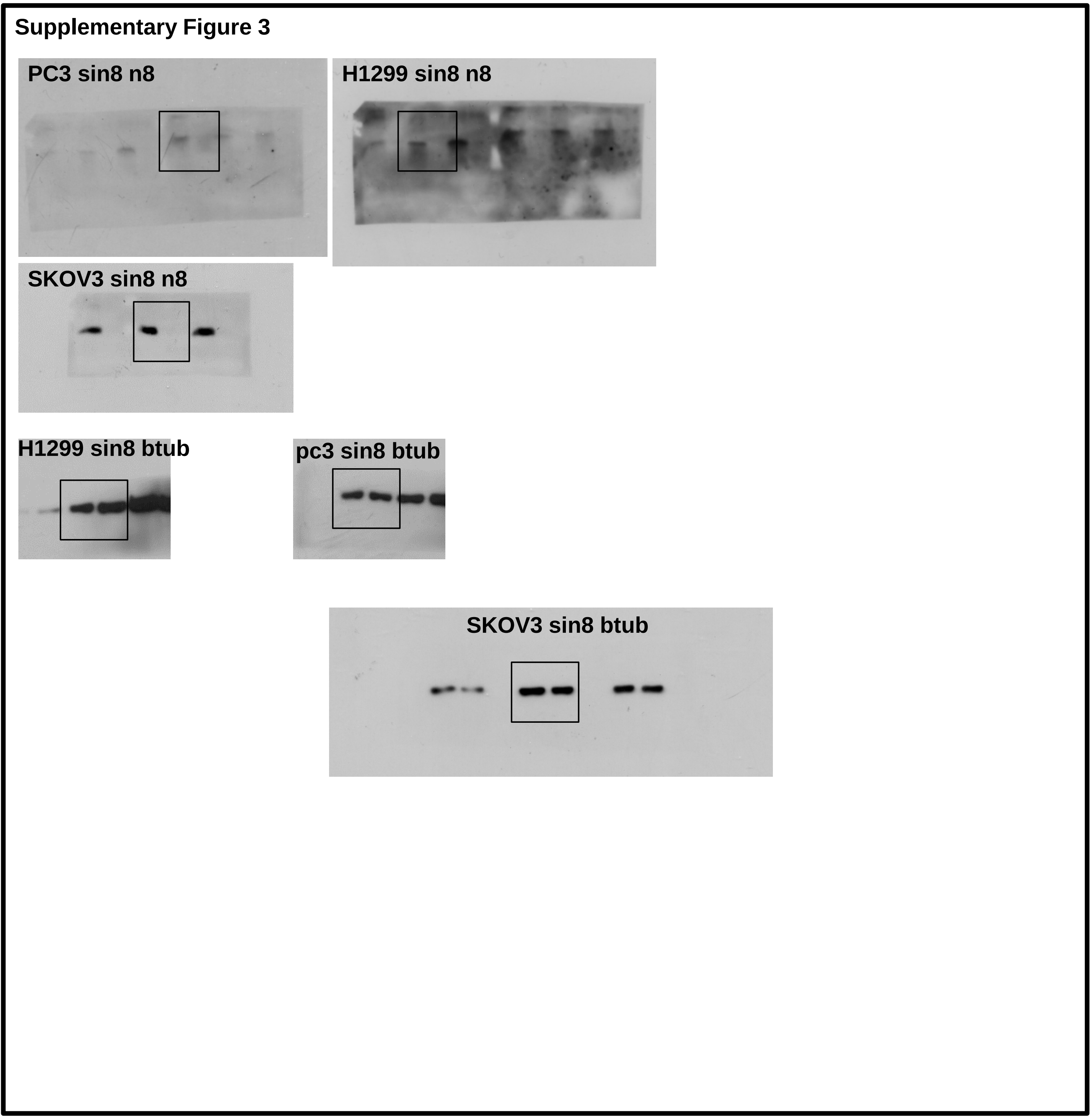

Supplementary Figure S8.

Supplementary Figure 5

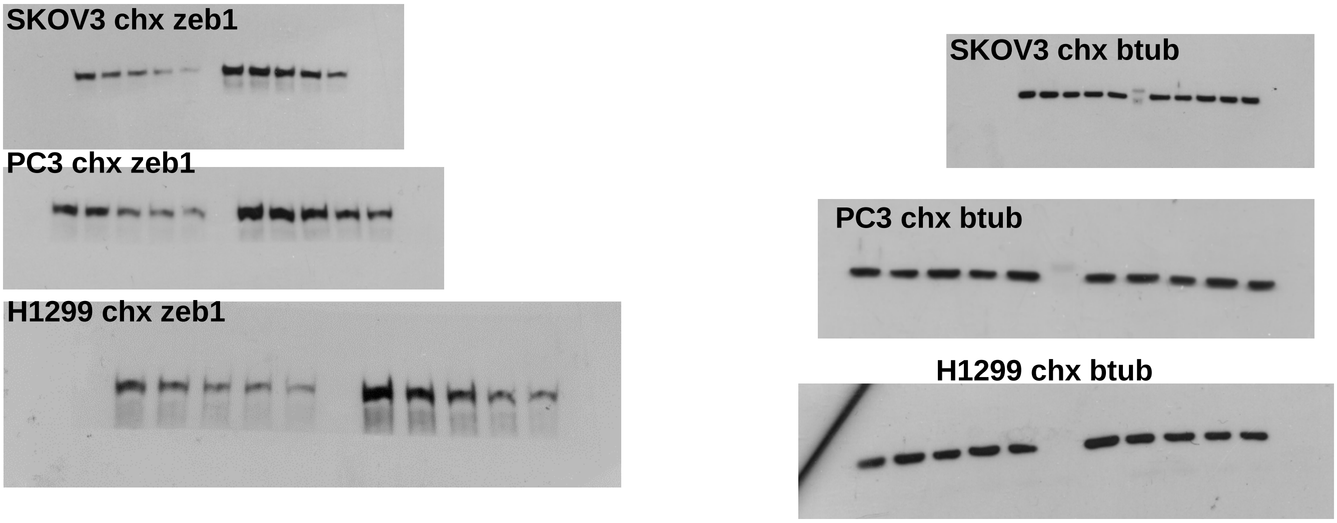

Supplementary Figure S8.

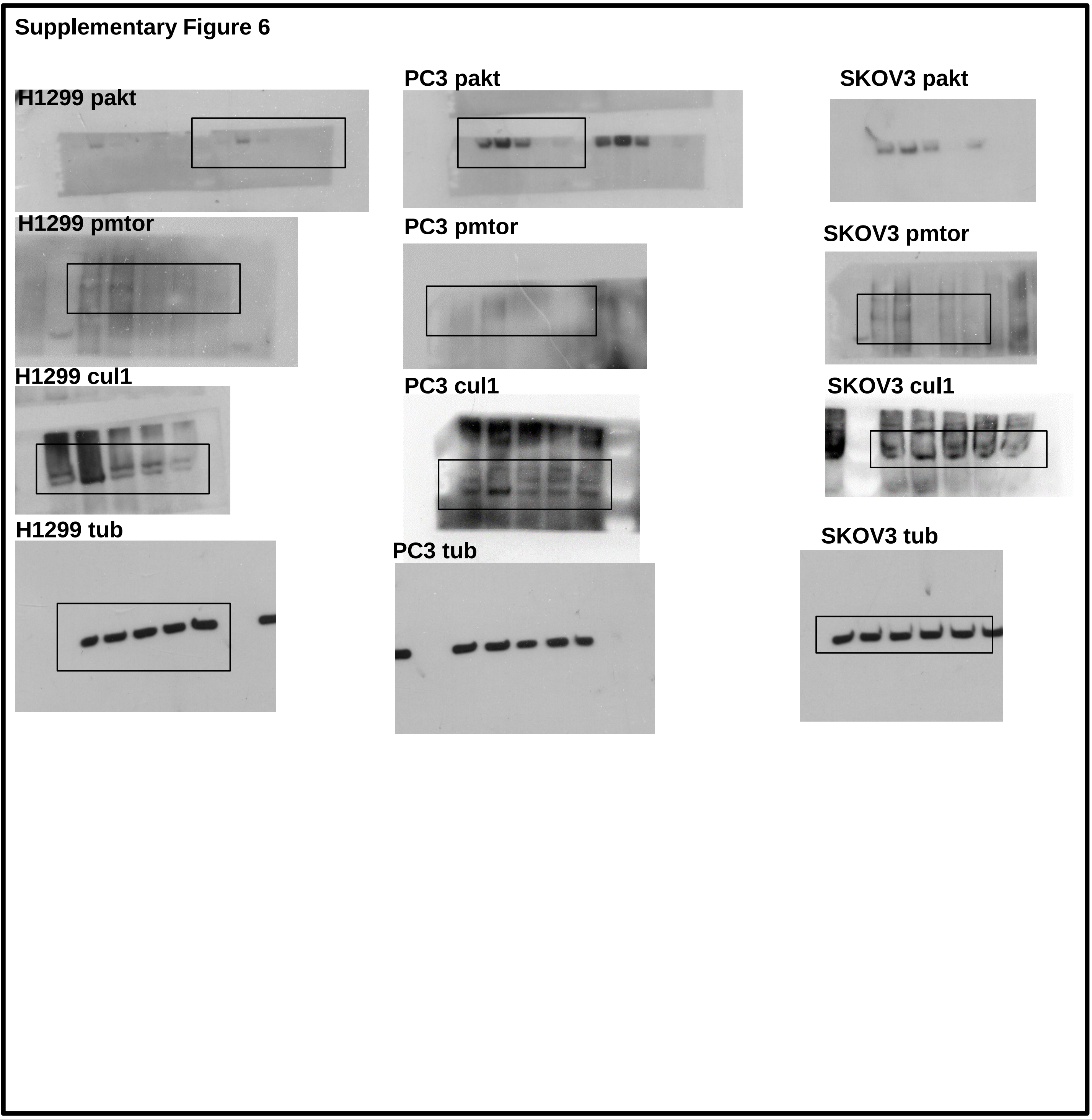

Supplementary Figure S8.

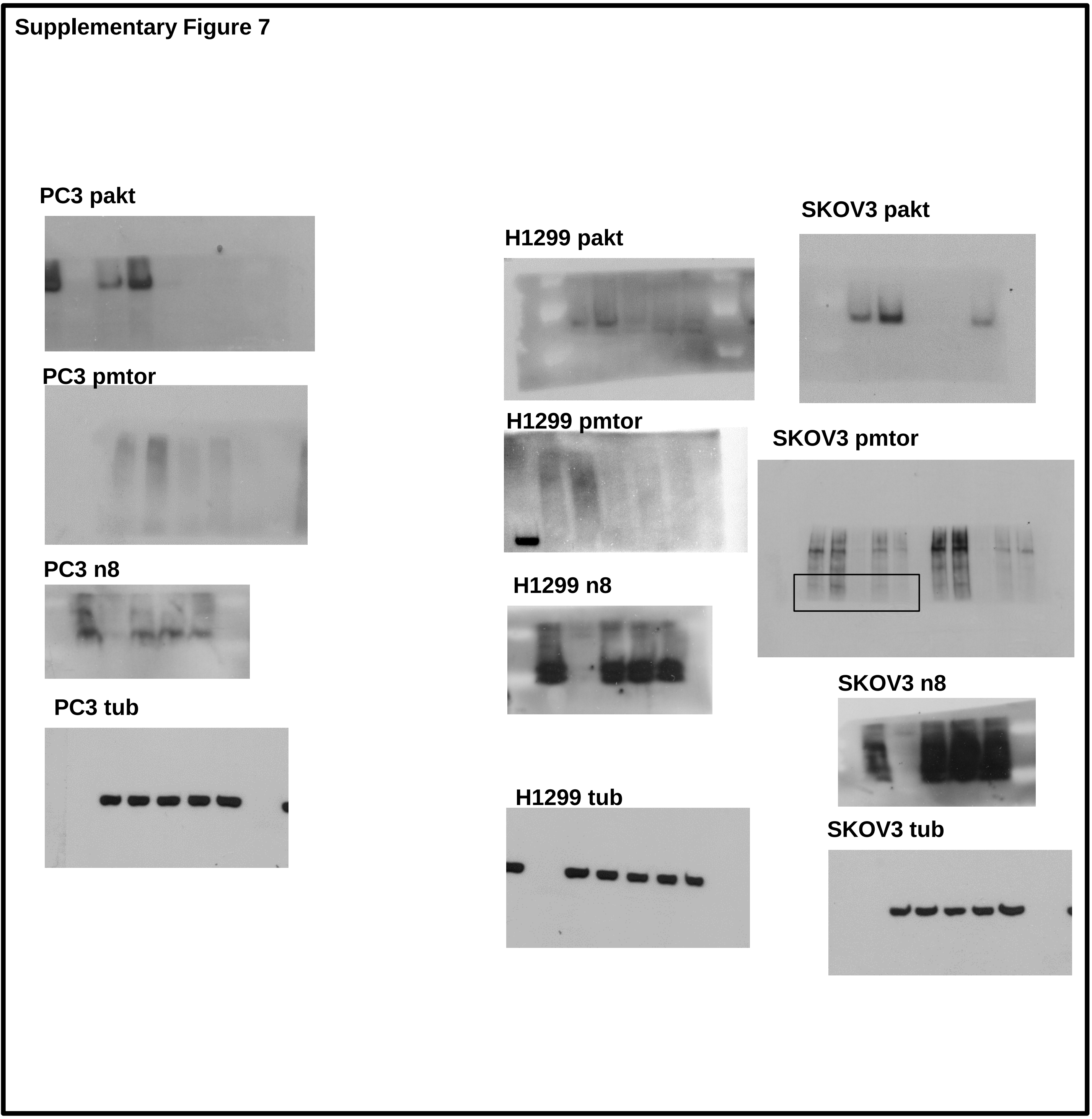

Supplementary Figure S9.

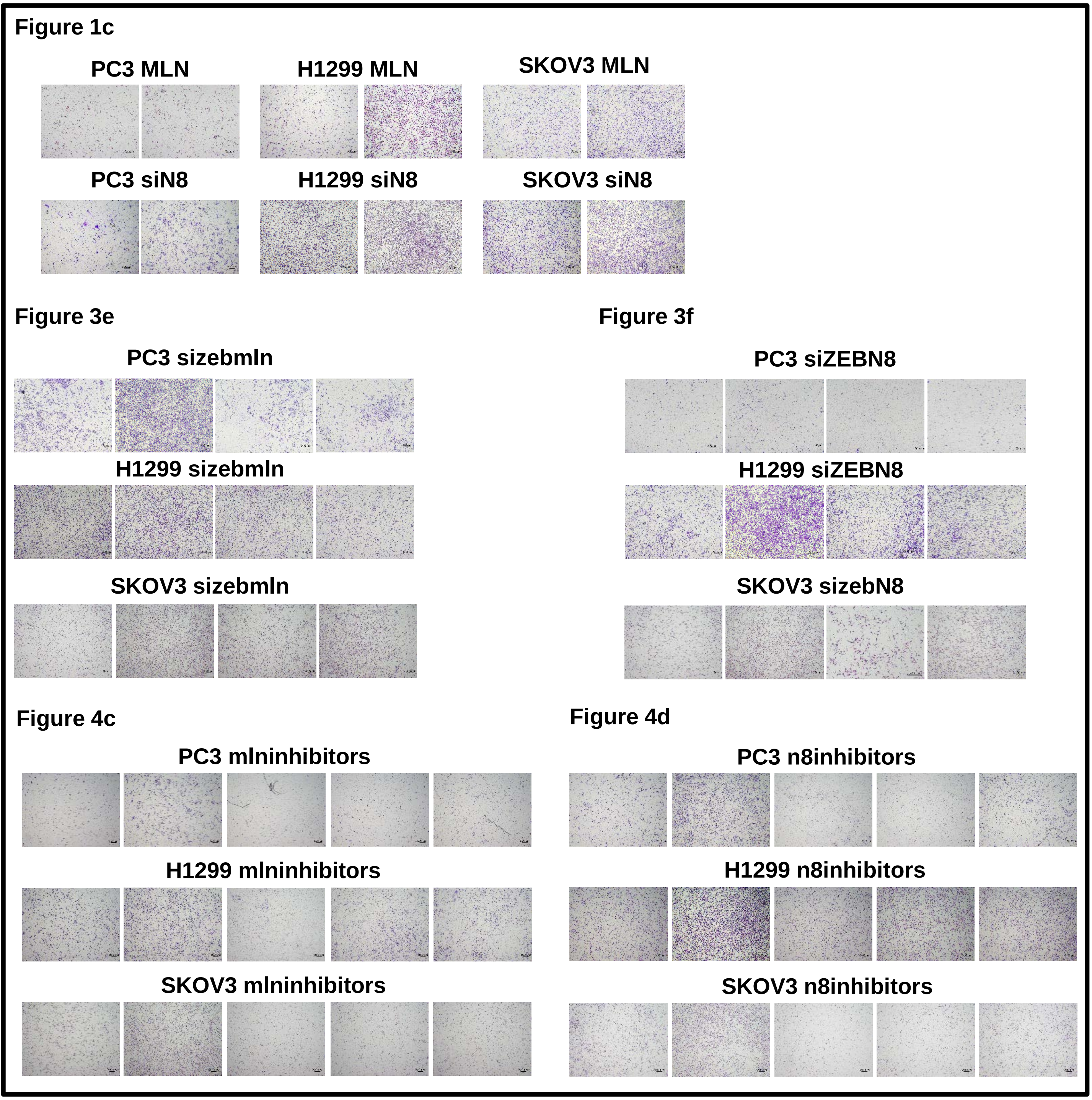

Supplementary Figure S9.

Figure 5e

PC3 sihifmln

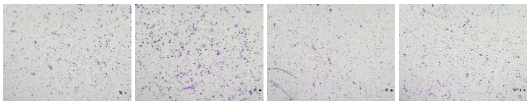

H1299 sihifmln

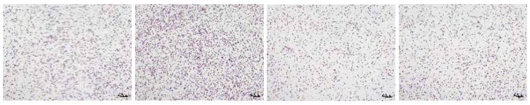

SKOV3 sihifmln

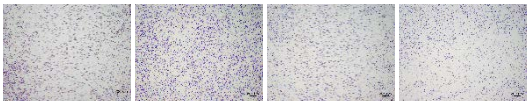

Figure 5f

PC3 siHIFN8

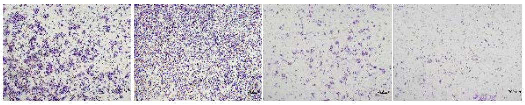

H1299 siHIFN8

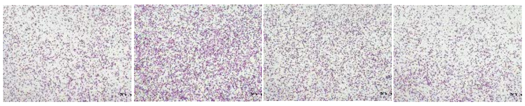

SKOV3 siHIFN8

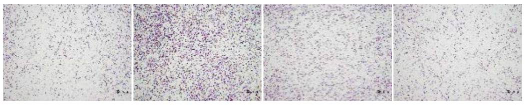

Supplementary Figure S3

PC3 sicn8

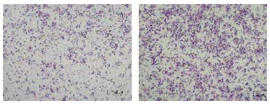

H1299 sicn8

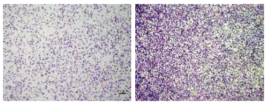

SKOV3 sicn8

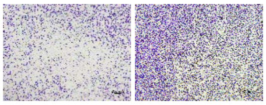

Supplement: Supplementary file 1 — Supplementary figures [file 41598_2020_75286_MOESM1_ESM.pdf]
