# Supplementary material for: Neddylation blockade induces HIF-1α driven cancer cell migration via upregulation of ZEB1
Source: Sci Rep. 2020 Oct 23;10:18210. doi: 10.1038/s41598-020-75286-0 (PMC7585416; doi:10.1038/s41598-020-75286-0)
Supplement: Supplementary file 2 — Supplementary tables [file 41598_2020_75286_MOESM2_ESM.docx]

Neddylation blockade induces HIF-1α driven cancer cell migration via upregulation of ZEB1

Jun Bum Park^1,2^, Jieun Seo^1,2^, Jong-Wan Park^1,2^ and Yang-Sook Chun^1,2,3,^*****

^1^Department of Biomedical Science, ^2^Ischemic/hypoxic disease institute, ^3^Department of Physiology, Seoul National University College of Medicine, Seoul 110-799, Korea

**Corresponding Author:** Yang-Sook Chun, Department of Physiology, Seoul National University College of Medicine, 103 Daehak-ro, Jongno-gu, Seoul 110-799, Republic of Korea. Phone: 82-2-740-8909; Fax: 82-2-3673-2167; E-mail: [chunys@snu.ac.kr](mailto:chunys@snu.ac.kr).

**Table S1. Information on the antibodies**

| **Primary antibodies** | **Supplier** | **Cat. No** |
| --- | --- | --- |
| NEDD8 | Cell Signaling Technology | 2745S |
| p-AKT (S473) | Cell Signaling Technology | 9271S |
| p-mTOR | Cell Signaling Technology | S2448 |
| Vimentin | Cell Signaling Technology | D21H3 |
| N-cadherin | Cell Signaling Technology | D4R1H |
| E-cadherin | Cell Signaling Technology | 24E10 |
| Slug | Cell Signaling Technology | C19G7 |
| CUL1 | Cell Signaling Technology | #4995 |
| mTOR | Cell Signaling Technology | 2983S |
| Akt (pan) | Cell Signaling Technology | #4691 |
| TCF8/ZEB1 | Cell Signaling Technology | D80D3 |
| β-tubulin | Santa Cruz Biotechnology | Sc9104 |

**Table S2. Information on si-RNAs**

Main figure N8 siRNA sequence: 1. 5’-AGCGGUA-GGAGCAGCAAUUUAUCCG-3’

Supplementary figure N8 siRNA sequence: 1. 5’–CCCUGGUUGUCAAUAAAAUAUUUCC-3’

ZEB1 siRNA sequence: 5’-GUUGGAGAAUAAUCAAGCCAAUCTT-3’

HIF1A siRNA sequence: 5’-GGGAUUAACUCAGUUUGAACUAACU-3’
